# Association of blood-based neurodegenerative biomarkers with cognitive functioning and dementia in India (LASI-DAD) and the United States (HRS)

**DOI:** 10.1093/aje/kwaf179

**Published:** 2025-12-02

**Authors:** Jung Ki Kim, Masroor Anwar, Abhishek Gupta, Bharat Thyagarajan, Peifeng Hu, Jessica D. Faul, David R. Weir, Kenneth M. Langa, Jinkook Lee, Sharmistha Dey, Eileen M. Crimmins

**Affiliations:** 1 Davis School of Gerontology, University of Southern California, Los Angeles, CA, United States; 2 Department of Biophysics, All India Institute of Medical Sciences, New Delhi, India; 3 Department of Laboratory Medicine and Pathology, University of Minnesota, Minneapolis, MN, United States; 4 Division of Geriatric Medicine, University of California, Los Angeles, Los Angeles, CA, United States; 5 Institute for Social Research, Survey Research Center, University of Michigan, Ann Arbor, MI, United States; 6 Division of General Medicine, Department of Internal Medicine, University of Michigan, Ann Arbor, MI, United States; 7 Institute of Gerontology, University of Michigan, Ann Arbor, MI, United States; 8 Department of Economics and Center for Economic and Social Research, University of Southern California, Los Angeles, CA, United States

**Keywords:** HRS, LASI-DAD, cognitive function, dementia, neurodegenerative biomarkers, cross-country comparison

## Abstract

This study examines associations between blood-based neurodegenerative biomarkers and cognitive functioning in nationally representative samples of older adults in India and the United States. Using data from the Longitudinal Aging Study in India-Diagnostic Assessment of Dementia (LASI-DAD) and the Health and Retirement Study (HRS), we analyzed 4 biomarkers—the ratio of Amyloid beta 42-40 (Aβ42/Aβ40), Glial Fibrillary Acidic Protein (GFAP), Neurofilament Light Chain (NfL), and Phosphorylated Tau at Threonine 181 (pTau181)—in relation to cognitive outcomes. Higher levels of GFAP and NfL were associated with worse cognitive function and a greater likelihood of dementia in both populations. GFAP and NfL were also associated with cognitive decline in the HRS, but not in LASI-DAD. Higher Aβ42/Aβ40 ratio was associated with worse cognitive functioning and more dementia in HRS; on the other hand, higher levels of the Aβ42/Aβ40 ratio were significantly linked with better cognitive functioning in LASI-DAD. pTau181 was generally not associated with cognitive functioning in either country. Our findings suggest few potential biomarker especially GFAP and NfL, for early manifestation of cognitive decline and dementia. The associations of the whole set of markers with subsequent mortality suggest they may serve as markers for general aging.

## Introduction

Cognitive decline and dementia are among the most pressing challenges facing aging societies worldwide. As global life expectancy continues to rise, the number of individuals with cognitive impairment and dementia will increase significantly, imposing substantial burdens not only on older people, but also on affected families, healthcare systems, and economies,^1^ thus making it a crucial area for public health intervention. Developing effective tools for the early detection of dementia is, therefore, critical.

Recent advances in biomarker research have identified blood-based neurodegenerative markers as efficient and practical tools for predicting cognitive impairment, cognitive decline, and the onset of dementia.^2^ These markers are particularly promising because of their potential for widespread use in clinical practice, overcoming the current limitations associated with costly amyloid positron emission tomography (PET) scans and cerebrospinal fluid (CSF) biomarkers. Blood-based tests are less invasive, more cost-effective, and easier to implement for large-scale screening and monitoring of neurodegenerative processes in diverse populations.^3–5^ They could change how we monitor progression of Alzheimer’s disease (AD) and treatment effects.^5^ Recent technological advancements have enhanced the sensitivity and specificity of blood-based biomarker assays, allowing for the detection of very low levels of neurodegenerative markers that were previously undetectable in plasma or serum.^6^ This has paved the way for the broader use of blood-based biomarkers in both clinical practice and epidemiological research across different countries.^7^

Amyloid beta 42 (Aβ42) and Amyloid beta 40 (Aβ40), often used as a ratio (Aβ42/Aβ40), Glial Fibrillary Acidic Protein (GFAP), Neurofilament Light Chain (NfL), and Phosphorylated Tau at Threonine 181 (pTau181) are among the blood-based neurodegenerative biomarkers now assayed in population studies where collection is done in the field rather than a clinic or laboratory. pTau181 is a biomarker of tau pathology, correlating with neurofibrillary tangles, a hallmark of AD.^8^ Elevated plasma pTau181 levels have been shown to predict future cognitive decline and proposed as a tool for diagnosis and prognosis of cognitive impairments and dementia, tracking progression and assessing treatment response.^3,7,9^ The Aβ42/Aβ40 ratio is indicative of amyloid plaque accumulation in the brain, also a hallmark of AD pathology. It is thought to serve as an early marker of amyloid pathology and is a reliable predictor of amyloid positivity as determined by PET scans.^10–14^ While a number of studies have reported that CSF Aβ42 concentrations are decreased with AD, studies generally report no change or higher plasma Aβ42 levels with AD.^15^ Recent studies provided additional clarity by demonstrating that decline in plasma Aβ42/Aβ40 ratio may occur prior to amyloid positivity on PET imaging (eg, PiB), potentially making this marker a useful tool for early diagnosis that could reduce the need for expensive PET scans in some cases.^16,17^ NfL, a marker of axonal damage and neurodegeneration, is elevated in several neurodegenerative diseases including AD. Higher plasma NfL levels are associated with greater disease severity, faster cognitive decline, and more pronounced brain atrophy.^11,18^ GFAP is recognized for its role in reflecting astrocytic activation and neuroinflammation.^17^ Elevated plasma GFAP levels have been linked to AD, particularly in individuals with early amyloid pathology but without significant tau deposition, suggesting that GFAP may serve as an early marker of amyloid-related neuroinflammation that could help identify individuals at risk of progressing to clinical stages of dementia.^19,20^

Given their broad applicability, these biomarkers are particularly valuable for cross-national research in populations with varying genetic, demographic, social, and environmental risk factors. India and the United States differ markedly in environmental, social, and economic factors that may contribute to effect modification on the associations between biomarkers and cognitive outcomes. For example, environmental exposures such as air pollution and poor water quality may disproportionately affect populations in India. Social and cultural differences, including education systems, societal roles of older adults, and levels of social engagement, may also shape cognitive aging differently. It is also true that lifestyle factors such as diet, physical activity, and access to healthcare differ significantly between the 2 countries. Economically, lifetime disparities in income, wealth distribution, and healthcare infrastructure are likely to influence both the levels of neurodegenerative biomarkers and their relationships to cognitive outcomes. The incorporation of neurodegenerative biomarkers into large-scale, population-based cohort studies is expected to significantly advance research on cognitive decline and dementia by indicating whether these biomarkers have similar utility in predicting cognitive outcomes in non-Western and Western contexts.^21,22^ Rigorous cross-laboratory validation procedures were done to ensure the reliability and comparability of the biomarker measurements across these 2 countries in order to minimize measurement artifacts and biases when making cross-country comparisons.^21–24^ Measurement of cognition using the Harmonized Cognitive Assessment Protocol (HCAP), which is implemented in both the Longitudinal Aging Study in India-Diagnostic Assessment of Dementia (LASI-DAD) and the Health and Retirement Study (HRS), further enhances the comparability of cognitive assessments across these contexts. Our expectation is that major differences in the exposures of the populations in the 2 countries to environmental, social, and economic risks may not only influence the levels of neurodegenerative biomarkers but also how these biomarkers relate to cognitive outcomes.

## Methods

### Study populations

This study uses data from 2 large nationally representative studies: the LASI-DAD and HRS. LASI is a survey of health, economic and social well-being of the population aged 45 and over in India; LASI-DAD is a representative subsample of LASI that has a focus on cognitive aging and dementia among persons aged 60 and over in India, with an oversampling of those at a higher risk of cognitive impairment.^22^ To measure cognition and dementia, LASI-DAD adopted an assessment protocol of the Harmonized Cognitive Assessment Protocol Project of the HRS (HRS HCAP) while considering India’s specific social and cultural context. A comprehensive overview of the study design and methods is available in Lee et al.^22,25^ We use data from LASI-DAD wave 1 collected between 2017 and 2019, and wave 2 collected in 2022-2024 with an average time of 4.5 years between the 2 waves.

The HRS is a longitudinal study of Americans over the age of 50 conducted biannually. The survey covers social, behavioral, and economic characteristics as well as physical and mental health. The HRS HCAP is a representative subset of the parent study, with extensive information on cognitive functioning and dementia that was initiated in 2016 with an extended cognitive protocol for HRS respondents aged 65 or older.^23^ The sample with neurodegenerative markers in HRS is a probability sample drawn from the 2016 venous blood study (VBS) sample who were eligible for the 2016 HCAP (aged 65 and over) or those younger than 65 who will be eligible for a future HCAP (a random half of HRS sample younger than age 65) (*n* = 4214).

Blood-based neurodegenerative biomarkers assayed from the HRS 2016 and LASI-DAD wave 1 data (2017-2019) are the focus of this paper. Those with information on blood-based neurodegenerative markers, cognitive functioning, and dementia were included in the analytic sample (see Figure S1). The sample with neurodegenerative biomarkers was 2669 in LASI-DAD and the final analytical sample consisted of 2151 participants with all 4 biomarkers available for analysis as well as the cognitive factor score, 1405 for dementia analysis, and 1128 for cognitive change analysis. Among those included at the first cognitive assessment, 496 participants died between waves (22.9%, weighted). The HRS sample was limited to those aged 60 and over (*N* = 3493) to match the age of the LASI-DAD sample. The final HRS sample included 3466 participants with all four neurodegenerative biomarkers, of whom 2333 were part of the HCAP sample and had a cognitive factor score, 2323 had HCAP data indicating whether they had dementia, and 2989 were included in the cognitive status change analysis. A total of 432 participants died in 4 years (11.2%, weighted).

### Measures

#### Neurodegenerative markers

Four biomarkers related to brain health and AD were examined: Aβ42/Aβ40 ratio, GFAP, NfL, and pTau181. These markers are plasma-based with the exception of HRS pTau181, which is serum-based. In addition, a summary biomarker factor score was developed using confirmatory factor analysis (CFA). Initially, all 4 neurodegenerative markers were included in the factor score model; but when Aβ42/Aβ40 was included, the measure did not converge and this measure was therefore omitted from the summary score. The final factor score was based on non-log-transformed GFAP, NfL, and pTAU181 for both LASI-DAD and HRS. The CFA factor loadings and fit statistics for neurodegenerative biomarker factors in LASI-DAD and HRS are provided in Table S1. There is no evidence that factor loadings differ significantly between HRS and LASI-DAD.

In India, trained phlebotomists collected blood samples from participants between 2017 and 2019. A total of 17 mL of blood was drawn, which was transported to local labs within 2 hours and processed to obtain serum and plasma and then shipped at 4 °C to the central laboratory in Delhi^26^; subsequently it was shipped to the AIIMS laboratory in Delhi and stored at −80 °C until the samples were assayed for the neurodegenerative markers in 2023 and 2024.

Assays for neurodegenerative markers were done in HRS after a pilot test to determine their validity and reliability using the HRS protocol for collection and overnight shipping to the laboratory.^27^ In 2016, phlebotomists collected blood samples for HRS at home visits and all tubes were shipped cold overnight to the University of Minnesota’s (UMN) Advanced Research and Diagnostic Laboratory arriving mostly within 24 hours (92% of the sample). The aliquoted samples were frozen on arrival and stored under −80 °C until assay was performed. Detailed description of the venous blood collection, laboratory equipment, and assay protocol has been reported previously by Crimmins et al.^28^

For the purpose of comparing cross-country associations between the neurodegenerative biomarkers and cognitive outcomes, lab approaches were harmonized across the studies. Results of the harmonization work indicated high correlation between the values obtained in the 2 labs; details are provided in the supplemental material (Appendix S1, Table S2, Figure S2a and b). In order to compare the relationships among biomarkers, compare across studies, and account for lab variability, biomarker values were standardized within each sample to transform the biomarkers to have a mean of 0 and an SD of 1 in our analyses.

#### Cognitive functioning and dementia

Cognitive functioning was assessed using a harmonized factor score developed for both LASI-DAD and HRS HCAP. This general cognitive factor score reflects the broadest cognitive summary variable including memory, executive functioning, visuospatial, and language domains.^29^

Dementia was defined in LASI-DAD using the consensus clinical dementia rating (CDR) which clinicians determined based on cognitive test results and informant reports using an online platform. Six cognitive and functional domains (memory, orientation, judgment and problem solving, community affairs, home and hobbies, and personal care) are the basis for the CDR. No impairment and questionable impairment in the CDR were coded as not demented while mild, moderate, and severe impairment as demented.^30^

In the HRS HCAP, dementia was defined through a 3-phase process involving the selection of a normative sample, standardization of cognitive test performance, and an algorithm-based classification.^31^ Dementia is defined by impairment in at least 2 cognitive domains along with informant-reported functional decline.^31^

#### Cognitive decline

For analysis of cognitive decline between the two waves, decline in the factor score between wave 1 and wave 2 was used for LASI-DAD. Because the factor score is not available for HRS at time 2 (2020), cognitive change is based on comparing the 2016 and 2020 Langa-Weir (LW) score of cognitive functioning.^32^ The change analysis was limited to those without dementia in the first wave (CDR-based dementia in LASI-DAD and LW classification-based dementia in HRS). Cognitive decline was defined as negative change from time 1 to time 2 in the factor score for LASI-DAD or LW cognitive score for HRS. Since people could die during the follow-up period, we included death as a separate outcome with cognitive decline.

#### Sociodemographic background

Sociodemographic factors such as age and sex, race/ethnicity, education, and rural residency may influence how these biomarkers are associated with cognitive outcomes.^33^ We divided the samples into 3 education categories that differed across both the countries. In LASI-DAD, low education was defined as never having attended school, midlevel education as having some primary or middle school, and high education as having completed middle school or higher. In HRS, low education was defined as having a high school education or less, midlevel education as having some college education but not graduating, and high education as having completed a college degree or higher.

### Statistical analysis

We show descriptive statistics at baseline in each sample; as well as the distribution of each marker in the 2 countries. Pearson’s correlations among the AD biomarkers in each country are also shown. We compared mean levels of each standardized biomarker by age to see if the biomarkers show expected differences. We examined the relationship of the neurodegenerative markers to cognitive functioning using ordinary least squares (OLS) regressions and logit regressions for dementia. In order to examine how neurodegenerative biomarkers are related to cognitive decline among those who did not have dementia in the first wave, we ran multinomial logistic regressions where the outcomes of cognitive decline and death were compared to no decline in cognitive function. We ran the models with each biomarker separately, and then with all biomarkers together since some studies in the literature show the predicted effect of combined biomarkers.^11^ For the cognitive change analysis, we present only the individual models since the results from the combined model were not different. We initially ran all models with controls for age, sex, and race/ethnicity in HRS, and age and sex in LASI-DAD. We also examined the associations with additional controls for education, caste, and living in a rural area for LASI-DAD and education for HRS. Since the inclusion of these factors did not change results in a meaningful way, we present the results from the more parsimonious model. In the supplementary analyses, we present the same multivariate results while controlling for assay plate. All statistical analyses were conducted using SAS and R, and statistical significance was defined as *P* < 0.05 in plots but the exact *P*-values are provided in the supplemental tables. Alzheimer’s disease biomarker weight for LASI-DAD and the VBS subsample weight for HRS were applied to all analyses.

## Results

Baseline characteristics of the LASI-DAD and HRS samples are presented in Table 1. The mean age of the participants from both the countries were similar, with LASI-DAD having a mean age of 69.6 years and HRS 70.5 years. A slightly higher percent of the HRS sample was female compared to the LASI-DAD sample (54.6% vs 50.2%). There were substantial differences in education levels between the two populations.

**Figure 1.**
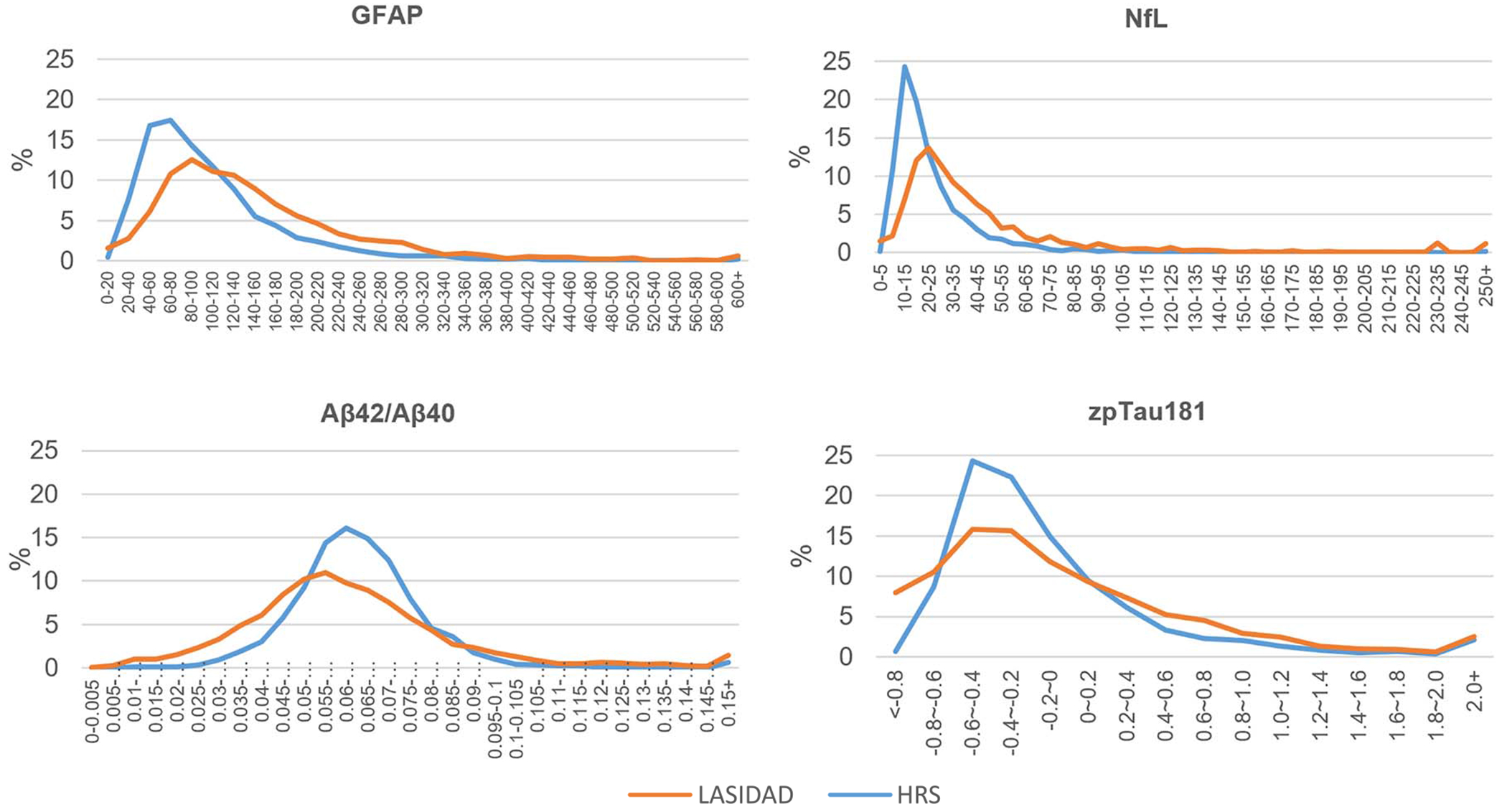
Distributions of neurodegenerative biomarkers in India (*N* = 2151) and the Unites States (*N* = 3466).

The LASI-DAD sample had higher median levels of GFAP (129.27 vs 90.30 pg/mL in HRS) and NfL (31.02 vs 18.60 pg/mL in HRS in Table 1). Higher levels of biomarkers among LASI-DAD were also evident in the distributions (Figure 1) where HRS tended to show a sharp peak at lower levels of GFAP, NfL, and pTau181 compared to LASI-DAD. The difference in the means of pTau181 is primarily due to the difference between plasma and serum values (Table 1).

**Figure 2.**
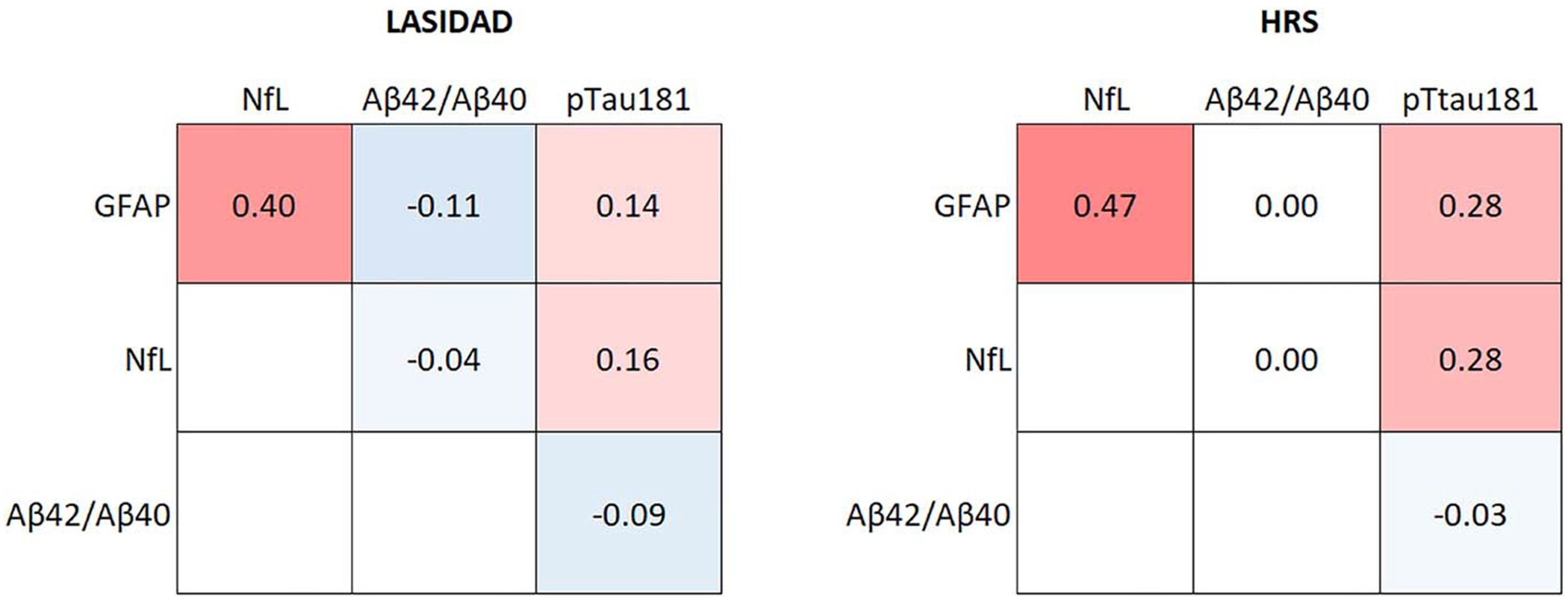
Correlations among neurodegenerative biomarkers in India (*N* = 2151) and the United States (*N* = 3466).

Correlations among GFAP, NfL, and pTau181 in the 2 countries were generally similar although somewhat stronger in HRS (Figure 2). Significant positive correlations were observed between GFAP and NfL (*r* = 0.40 for LASI-DAD; *r* = 0.47 for HRS), and the correlation between GFAP and pTau181 was 0.14 in LASI-DAD and 0.28 in HRS and that of NfL with pTau181 was 0.16 for LASI-DAD and 0.28 for HRS. On the other hand, a negative correlation of GFAP and NfL with Aβ42/Aβ40 was observed in LASI-DAD but not in HRS.

**Figure 3.**
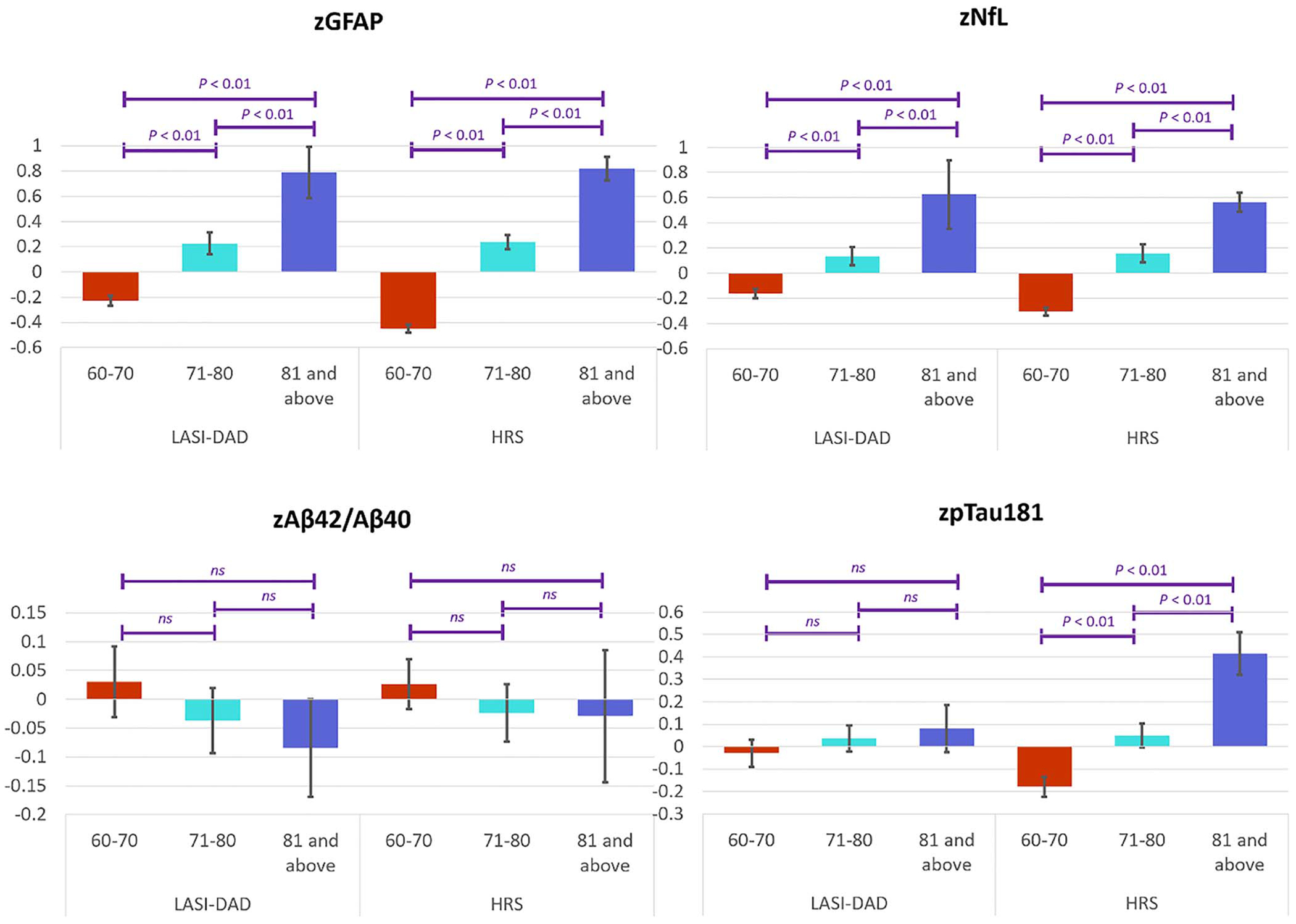
Standardized neurodegenerative biomarker levels by age in LASI-DAD (*N* = 2151) and HRS (*N* = 3466).

Given that age can influence biomarker levels and their impact could differ in two populations, we examined the variation in these biomarkers by age in LASI-DAD and HRS (Figure 3). The levels of GFAP and NfL were significantly higher at older ages in both the countries, while there was no difference in the level of Aβ42/Aβ40 among different age groups in either LASI-DAD or HRS. The level of standardized pTau181 was significantly higher at older ages in HRS, but not in LASI-DAD.

**Figure 4.**
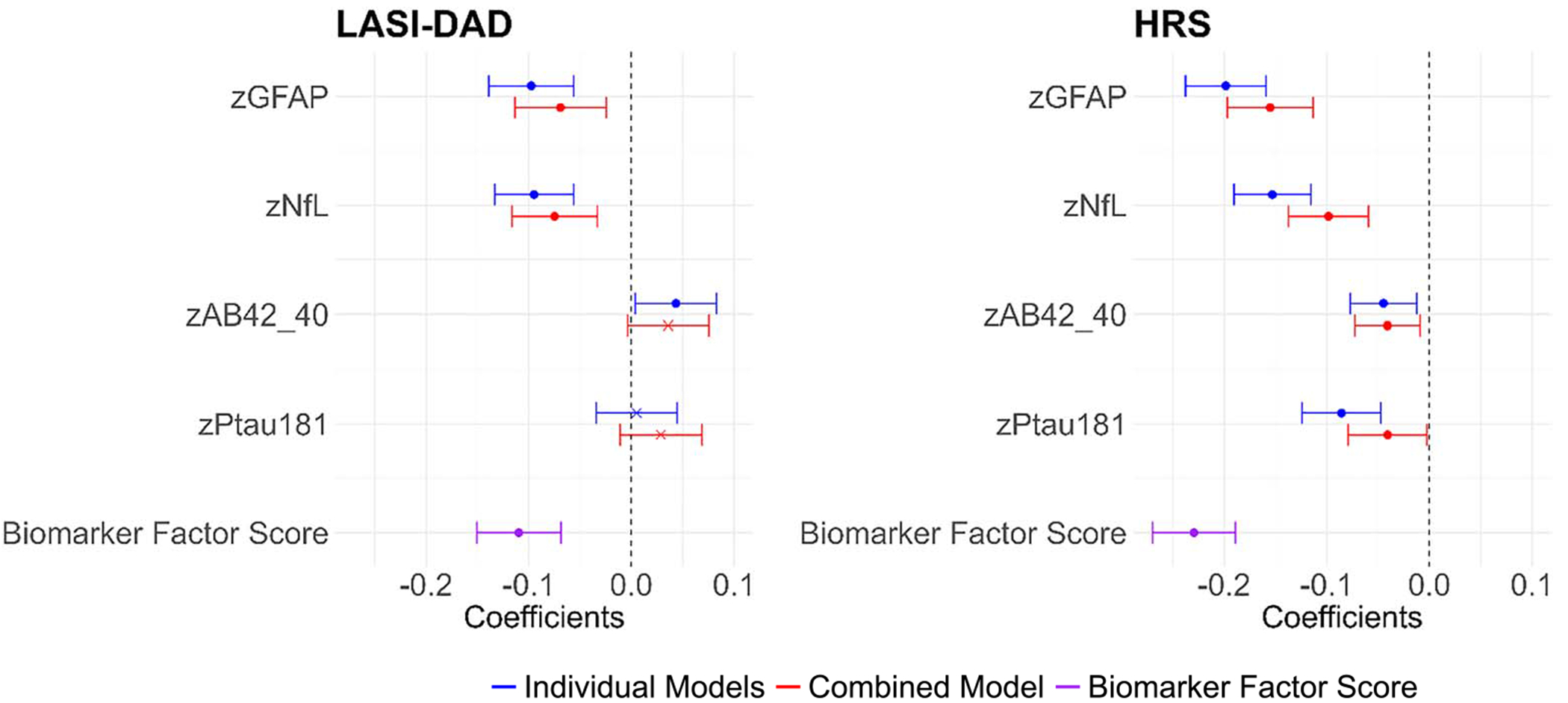
Association of standardized factor cognitive score on neurodegenerative biomarkers in India (*N* = 2151) and the United States (*N* = 2323). Note: Regression coefficients from OLS regression of standardized cognitive score on standardized GFAP, NfL, Aβ42/Aβ40, and pTau181 or neurodegenerative biomarker factor score (based on GFAP, NfL, pTau181); HRS: Age-, gender-, race/ethnicity-controlled; LASI-DAD: Age-, gender-controlled. Note: Individual models show when each biomarker was included in the model separately; combined model shows when all biomarkers were entered at the same time; biomarker factor score shows when biomarker factor score was included in the model. Note: Coefficients, 95% CI, and *P*-values are included in Table S3. *P* < 0.05.

The associations between neurodegenerative biomarkers and cognitive functioning are shown in Figure 4 (all numeric details are provided in Table S3). Two indicators of the association are shown for each marker: blue indicates the effect with 1 neurodegenerative marker in the equation and red indicates the association with all neurodegenerative markers in the equation. Associations are from regression equations, with age, sex, and race/ethnicity (HRS only) included as controls. For both GFAP and NfL, the direction of association was the same in LASI-DAD and HRS: higher levels of GFAP and NfL were associated with poorer cognitive functioning in both studies (for individual models, *b* = −0.10, in LASI-DAD; *b* = −0.20, in HRS for GFAP; *b* = −0.09, in LASI-DAD; *b* = −0.15, in HRS for NfL, with similar, but lower, coefficients for combined models). Lower Aβ42/Aβ40 was related to better cognitive functioning in HRS; in the LASI-DAD individual marker model, but not combined model, higher Aβ42/Aβ40 was associated with a better cognitive score. A higher pTau181 was related to worse cognitive functioning in HRS, but not in LASI-DAD. A higher summary biomarker factor score was related to a lower cognitive score for both LASI-DAD and HRS.

**Figure 5.**
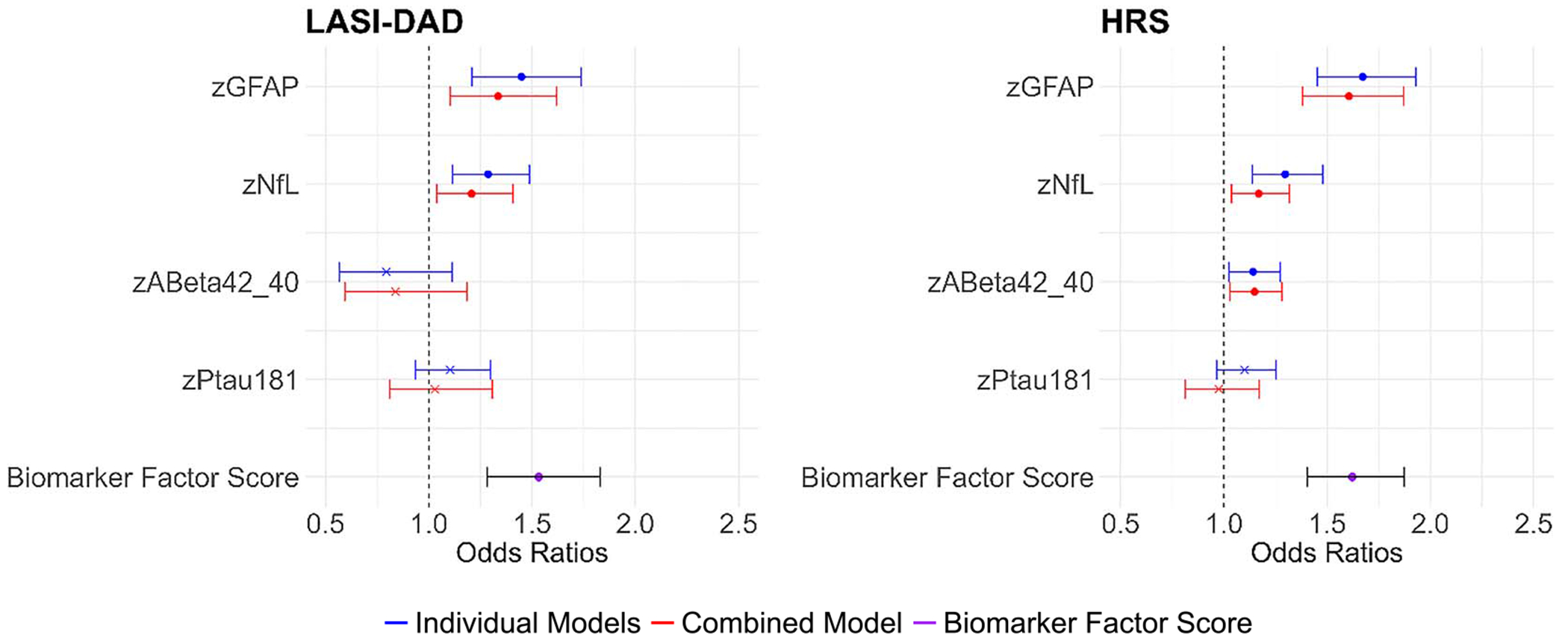
Association of dementia with neurodegenerative biomarkers in India (*N* = 1405) and the United States (*N* = 2333). Note: Odds ratios from logistic regression of having “dementia” on standardized GFAP, NfL, Aβ42/Aβ40, and pTau181 or neurodegenerative biomarker factor score; HRS: Age-, gender-, race/ethnicity-controlled; LASI-DAD: Age-, gender-controlled. Note: Individual models show when each biomarker was included in the model separately; combined model shows when all biomarkers were entered at the same time; biomarker factor score shows when biomarker factor score was included in the model. Note: Odds ratios, 95% CI, and *P*-values are included in Table S4. *P* < 0.05.

We found fairly similar results for GFAP and NfL in the association of the neurodegenerative biomarkers with dementia in both the countries; those with higher levels of GFAP and NfL were more likely to have dementia both in the individual models and the combined models in both countries (Figure 5, Table S4). A higher standardized GFAP increased the relative likelihood of having dementia by 45% for the individual model in LASI-DAD and 67% for the individual model in HRS. The Aβ42/Aβ40 ratio was not significantly related to having dementia in LASI-DAD, but a higher Aβ42/Aβ40 was associated with an increased likelihood of having dementia (14%-15%) in HRS. pTau181 was not related to dementia in either country. A higher combined biomarker factor score was associated with an increased likelihood of having dementia in both LASI-DAD and HRS.

**Figure 6.**
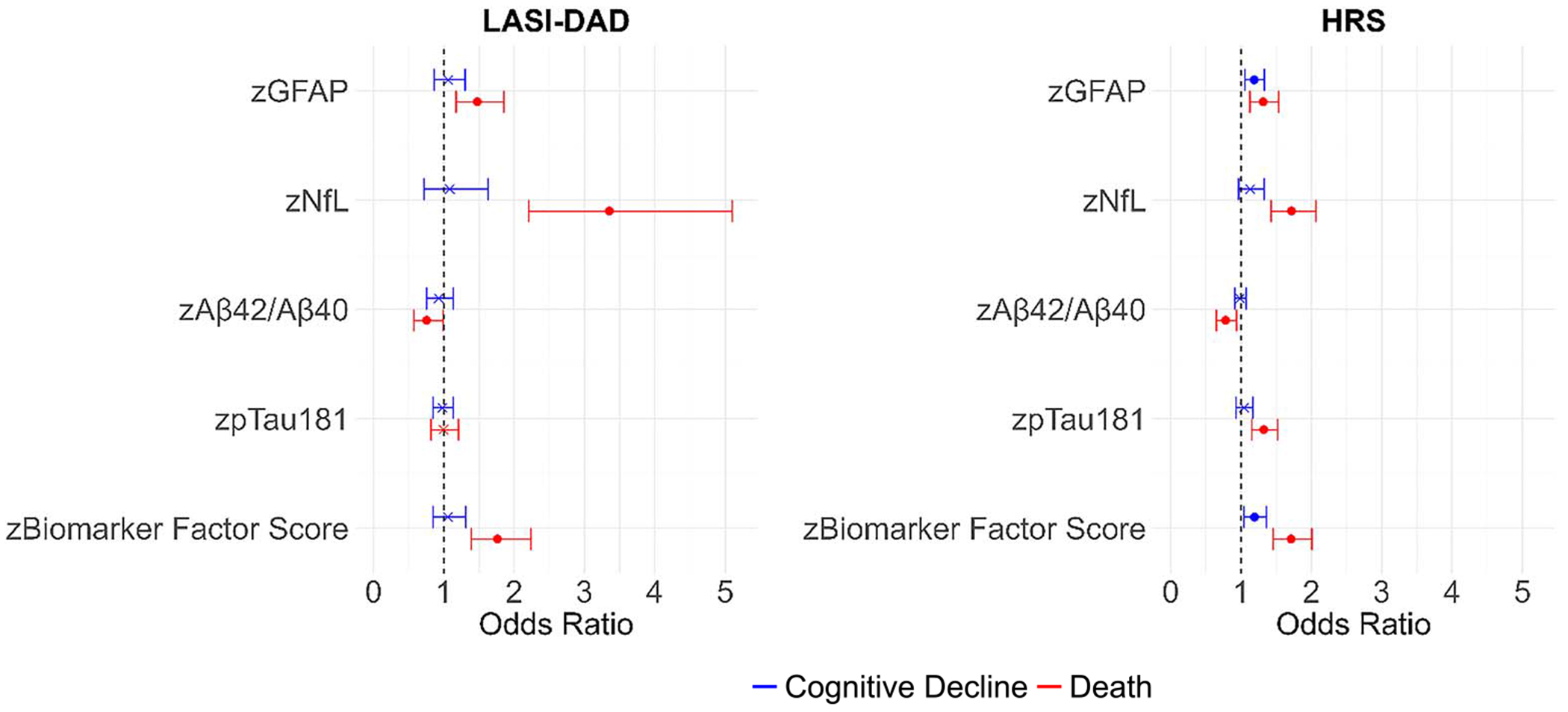
Association of cognitive decline and death with neurodegenerative biomarkers in India (*N* = 1128) and the United States (*N* = 2989). Note: Cognitive decline is defined as having a negative change between time 1 and time 2 cognitive score among those without dementia at time 1. Note: Each biomarker was included in the model separately. Note: Odds ratios, 95% CI, and *P*-values are included in Table S5. *P* < 0.05.

While some neurodegenerative markers were shown to be cross-sectionally related to cognitive function and dementia in the two countries, how those markers relate to cognitive change may not show the same pattern. Multinomial logistic regression results for cognitive decline with a competing risk of death, are presented in Figure 6 and also in Table S5. There are different associations of neurodegenerative biomarkers with cognitive decline in LASI-DAD and HRS. In LASI-DAD, none of the markers including the biomarker factor score was associated with cognitive decline. In HRS, higher GFAP levels (OR = 1.19) and a higher factor score (OR = 1.19) were related to an increased likelihood of cognitive decline compared to no cognitive change in HRS. We should note that all markers (higher GFAP, higher NfL, lower Aβ42/Aβ40, and higher pTau181) were associated with a greater likelihood of death in HRS; and all except pTau181 were associated with higher likelihood of death in LASI-DAD.

## Discussion

In this study, we examined the associations between blood-based neurodegenerative biomarkers and cognitive outcomes in two nationally representative samples of populations living under quite different circumstances—India and the United States. Overall, the results indicated that the associations between some neurodegenerative biomarkers—GFAP and NfL—and cognitive outcomes were in the expected direction and fairly consistent across the LASI-DAD and HRS samples when analyzed cross-sectionally. The findings that higher levels of GFAP and NfL were strongly associated with poorer cognitive functioning and an increased likelihood of dementia in both populations suggest that despite the differences in genetic background, environmental exposures, economic development, lifestyle factors, and healthcare systems between the two countries, these biomarkers are robust indicators of cognitive function and dementia.

While our regression models controlled for age and sex, and additionally for race/ethnicity for HRS, the results were consistent even after controlling for education, caste, and rural residency in India and for education in the United States (Tables S6–S8). This suggests that these biomarkers’ associations with cognitive outcomes may not be confounded by socioeconomic status and reinforces the potential utility of these neurodegenerative markers, particularly GFAP and NfL, as key biomarkers in predicting cognitive decline and dementia across diverse populations.

However, the similarities of the associations of biomarkers with cognitive outcomes in cross-sectional relationships did not extend to the ability of these biomarkers to predict cognitive change over the time observed here. While higher levels of GFAP and a summary biomarker factor score based on three of the neurodegenerative biomarkers were related to cognitive decline in the HRS, these associations were not observed in LASI-DAD. This inconsistency may be due to the difference in sample size between the countries, or the demographic and environmental variation between the populations, which could influence the progression of neurodegeneration and its impact on cognitive outcomes over the time. The differential impact observed in two countries could result from varying capacities to cope with neurodegenerative changes as people age, potentially due to differences in cognitive reserve developed over time.^34,35^ Additionally, cognitive decline in our analysis was based on different measures in LASI-DAD and HRS. To check whether the results were affected by having measures that are on different scales, we conducted a sensitivity analysis by transforming the LW scores into the same scale as LASI-DAD’s factor score, and found that most findings (except NfL) remained the same as the original results (Table S9). The absolute magnitude of cognitive change is similar in LASI-DAD (mean = −0.1, SD = 0.5, range: −2.5 to 2.0) and the HRS when transformed to LASI-DAD scale (mean = −0.1, SD = 0.9, range: −3.5 to 5.0) although the range is much wider in HRS. Along with that, these biomarkers may be more strongly associated with cognitive decline when baseline cognitive function is relatively high, but they may not be as predictive when baseline cognitive function is low.

Interestingly, while GFAP and NfL emerged as strong predictors of cognitive outcomes, the Aβ42/Aβ40 ratio and pTau181 was not found to be significant as was hypothesized. The neurodegenerative markers such as GFAP and NfL appear to be quitepromising. The Aβ42/Aβ40 showed only modest associations with cognitive outcomes, particularly in LASI-DAD, where the associations were relatively small and, in HRS, the associations were less consistent. Recent studies have suggested the plasma pTau181/Aβ42 ratio is a potentially sensitive marker of brain pathology.^36–41^ We tested whether this measure combining amyloid and tau levels could provide insights into the relationship between neurodegenerative markers, particularly Aβ42/Aβ40, and cognitive score. Interestingly, the unexpected direction of the relationship was observed between pTau181/Aβ42 and the cognitive score (ie, lower pTau181/Aβ42 ratio is related to a higher cognitive score) mirrored the pattern previously observed between Aβ42/Aβ40 and cognitive score. Further studies would be necessary to better understand the underlying mechanisms driving this unexpected association.

Furthermore, It is interesting to note that when we controlled for plate, there were very few changes from the analysis presented above, but the most frequent were the changes in the significance of Aβ42/Aβ40 (see Tables S10–S12). pTau181, on the other hand, did not show the expected associations in either sample, suggesting potential variation in the quantitative i measurement. Several potential measurement issues can be noted. First, in HRS, serum rather than plasma was used as the source for pTau181. Serum and plasma are not always comparable across different biomarkers, which could lead to variability in results for pTau181. Since the field conditions for LASI-DAD and HRS are very different, preanalytic factors such as sample handling, processing times, and storage conditions can introduce variability inspite of following all the preanalytical guidelines.

The fact that these biomarkers relate to mortality in both samples may indicate that they really are markers of general “aging” and not just indicators of neurodegeneration. Further work should follow up on how these neurodegenerative makers are associated with other markers of general aging such as epigenetic age and biological age. This highlights the complexity of cognitive aging and suggests that while neurodegenerative biomarkers are important, other factors, such as genetic predisposition and cognitive reserve developed by stimulating lifestyle and environmental factor, better education and occupation, that play significant roles in cognitive decline and dementia, should be explored together in future research.

This study has some limitations that should be noted. First, as mentioned, while we attempted to standardize biomarker measurements in the samples across the two countries, differences in laboratory procedures, sample handling, and assay sensitivity may have introduced variability that could affect the comparability of the results. However, when applying the harmonization equations among the two laboratories (Table S2), similar mean values were observed for original and harmonized levels of the biomarkers. The relatively short follow-up period (4.5 years, on average, in LASI-DAD and 4 years in HRS) might have constrained our ability to detect longer term cognitive changes associated with neurodegenerative biomarkers.

The findings from this study point to several important directions for future research on neurodegenerative markers and cognition in an international context. First, there may be a need to improve the measurement and standardization of biomarkers like Aβ42/Aβ40 and pTau181 to ensure their reliability and validity across different populations. This could involve developing more sensitive assays and conducting further cross-laboratory validations to address potential measurement issues. Second, future studies should focus on longitudinal analyses that track the progression of neurodegeneration over time in diverse populations. Both HRS and LASI-DAD will soon release the second wave of neurodegenerative marker data, which will help clarify the predictive value of these biomarkers for cognitive decline and incidence of dementia, and determine how factors like genetics, environment, and healthcare access interact with these biomarkers to influence cognitive change over time. Finally, expanding data collection to include other promising neurodegenerative biomarkers (eg, pTau 217)^42^ may improve our understanding of the pathways linking neurodegenerative markers to future cognitive outcomes and dementia.

## Supplementary Material

supplementary material

Supplementary material is available at the *American Journal of Epidemiology* online.

## Figures and Tables

**Table 1.** Description of the samples.

	LASI-DAD *N* = 2151	HRS *N* = 3466
Mean (SD) age (years)	69.6 (7.4)	70.5 (8.6)
Female (%)	50.2	54.6
Race/ethnicity (%)		
Non-Hispanic White/other	NA	81.4
Non-Hispanic Black	NA	9.6
Hispanic	NA	9.0
Education (%)		
Low education	52.0	14.7
Mid education	25.7	30.3
High education	22.3	55.0
Rural residency (%)	69.9	NA
Mean (SD)/Median neurodegenerative marker		
GFAP^a^	153.92 (107.40)/129.27	109.37 (77.38)/90.30
NfL^a^	44.38 (55.76)/31.02	25.48 (26.27)/18.60
Aβ42/Aβ40	0.06 (0.04)/0.06	0.07 (0.02)/0.06
pTau181	41.21 (29.46)/35.20	2.18 (2.27)/1.64
zpTau181	−0.00 (0.96)/−0.20	−0.05 (0.90)/−0.26
Mean (SD)/Median biomarker factor score (from GFAP, NfL, pTau181)^a^	1.33 (25.49)/−4.80	−0.02 (14.65)/−4.16
Cognitive measures		
Mean (SD) cognitive score		
Cognitive factor score	−0.07 (0.91)	0.23 (0.92) *N* = 2323
Dementia		
Demented (CDR) (*N* = 1405) (%)	6.7	
Demented (HCAP) (*N* = 2333) (%)		6.8
Cognitive change	*N* = 1128	*N* = 2989
No cognitive decline (%)	29.7	43.4
Cognitive decline (%)	48.4	45.3
Death (%)	21.9	11.3

a Mean country difference at *P* < .05;

Note: NA - Not applicable.

## Data Availability

All data relevant to the study are included in the article or uploaded as supplementary information.
