# Supplementary material for: Association of blood-based neurodegenerative biomarkers with cognitive functioning and dementia in India (LASI-DAD) and the United States (HRS)
Source: Am J Epidemiol. 2025 Aug 21;194(12):3705–13. doi: 10.1093/aje/kwaf179 (PMC12497661; doi:10.1093/aje/kwaf179)
Supplement: Web_Material_kwaf179 [file web_material_kwaf179.zip › supplement FINAL.docx]

**Supplemental Material**

**Association of Blood-based Neurodegenerative Biomarkers with Cognitive Functioning and Dementia in India (LASI-DAD) and the United States (HRS)**

Jung Ki Kim, Masroor Anwar, Abhishek Gupta, Bharat Thyagarajan, Peifeng Hu, Jessica D. Faul, David R. Weir, Kenneth M. Langa, Jinkook Lee, Sharmistha Dey, Eileen M. Crimmins

Figure S1. Sample Chart

Appendix S1. Harmonization of the Neurodegenerative Assays across Two Labs

Figure S2. a. Assay Validation in at AIIMS Delhi Laboratory

Figure S2. b. Eliminating the 3 Points for NfL and the 1 Point for pTau181 that are Clearly Off the Line

Figure S3. Distributions of Harmonized Neurodegenerative Biomarkers in India (N=2,151) and the US (N=3,466)

Table S1. Harmonization Results for LASI-DAD Sample between AIIMS Delhi Laboratory and the University of Minnesota

Table S2. CFA Factor Loadings and Fit Statistics for Neurodegenerative Biomarker Factors in LASI-DAD and HRS

Table S3. Coefficients for Figure 4

Table S4. Odds Ratios for Figure 5

Table S5. Odds Ratios for Figure 6

Table S6. Coefficients for the Results on Cognitive Function in Figure 4 and Table S2 (yellow indicates change in significance) when Caste, Rural Residency (LASI-DAD only) and Education are Controlled

Table S7. ORs for the Results on Dementia in Figure 5 and Table S3 (yellow indicates change) when Caste, Rural Residency (LASI-DAD only) and Education are Controlled

Table S8. ORs for the Results in Figure 6 and Table S4 (yellow indicates change) when Caste, Rural Residency (LASI-DAD only) and Education are Controlled

Table S9. ORs for the Results in Figure 6 based on Transformed HRS Scale for HRS

Table S10. Coefficients for the Results on Cognitive Function in Figure 4 and Table S2 (yellow indicates change in significance) when Plates is Controlled

Table S11. ORs for the Results on Dementia in Figure 5 and Table S3 (yellow indicates change) when Plate is Controlled

Table S12. ORs for the Results in Figure 6 and Table S4 (yellow indicates change) when Plate is Controlled

**Figure S1. Sample Chart**


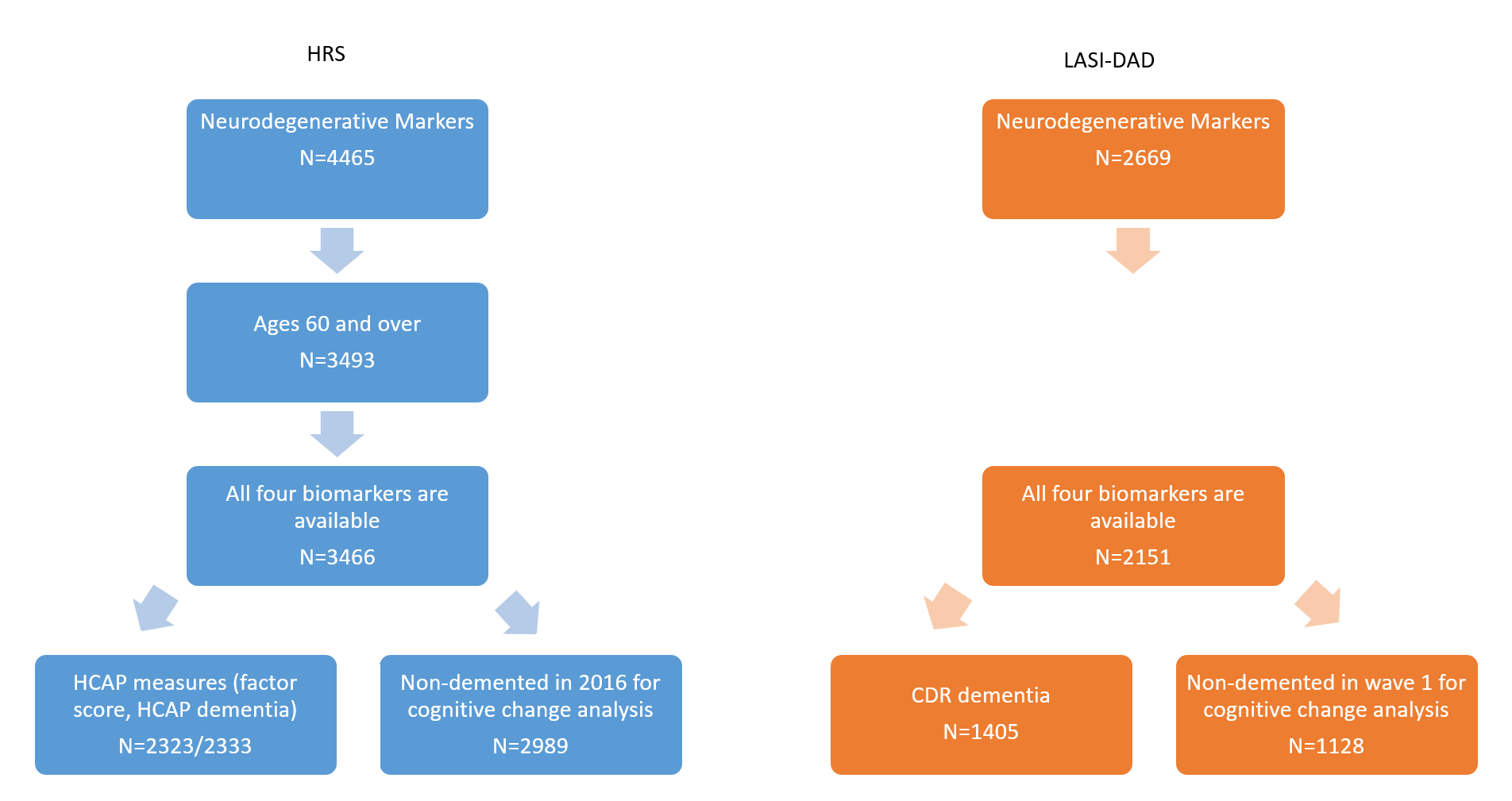


**Appendix S1. Harmonization of the Neurodegenerative Assays across Two Labs**

Neurodegenerative markers for the 2016 HRS sample were assayed in the Advanced Research and Diagnostic Laboratory (ARDL) in the Department of Laboratory Medicine and Pathology at the University of Minnesota in 2022 and 2023. These markers were done after a pilot test to determine the validity and reliability of neurodegenerative assays collected in the field under the HRS protocol and shipped overnight to the laboratory. The details and results of this initial pilot are provided in Panikkar et al. 2023.^25^

In order to ensure comparability across countries for the neurodegenerative markers, the LASI-DAD lab members were trained on the instrument used by HRS at the UMN. The same Quanterix HDX Simoa analyzer, a highly sensitive immunoassay platform, was purchased for use at AIIMS Delhi laboratory and a comparison of assay results was conducted from duplicate plasma specimens prepared and assayed in Minnesota and in Delhi before beginning the analysis of study samples.

The harmonization of assay preparation for HRS and LASI-DAD was accomplished through training of AIIMS laboratory staff from Delhi at the University of Minnesota laboratory and purchase of an identical machine for use at AIIMS, a Quanterix HDX fully-automated SIMOA analyser. After training, a total of 81 duplicate plasma samples were plated at Minnesota and send via a cold chain to AIIMS. These were then assayed for Aβ42, Aβ40, NFL, GFAP, t-tau and pTau181 at the two labs to compare results. The laboratories were blinded to the results to avoid bias during the analysis.

The results of the assays from the two labs are shown below in Figure S2. a and b. There is a very similar ranking of the assay values from the two labs with correlation coefficients ranging from 0.88 for NfL to 0.98 for Aβ40 (Figure S2.a). NfL and pTau181 have relatively low R2. In order to get a better equation for harmonization, we recompute the equation eliminating the 3 points for NfL that are clearly off the line and the 1 point for pTau181 that is clearly off the line. We assumed that these off-diagonal points represented random technical errors either at UMN or at the AIIMS laboratory and so these data points would not contribute meaningfully to the comparison of assays across the two laboratories. When 3 cases that are obviously out of line are eliminated from NfL (Figure S2.b), the correlation is .98; for pTau181the correlation increases to 0.89. This makes it clear that the labs are producing similar assay results and can be compared. We should note that there is some difference in level which results in our using z scored measures in the analysis. It is also possible to use the equations in table S2 to create harmonized values for the two labs. We do this by inserting the assayed value for LASI-DAD into the equation below and estimate the value for LASI-DAD in University of Minnesota values. The effect of doing this is show in Table S1 which compares the original means and the harmonized means for LASI-DAD and in Figure S3 which compares the distribution of harmonized values for LASI-DAD and HRS.

Panikkar D, Vivek S, Crimmins E, et al. Pre-analytical variables influencing stability of blood-based biomarkers of neuropathology. J Alzheimers Dis. 2023;95(2):735-748. doi: 10.3233/JAD-230384.

**Figure S2.**

**a. Assay Validation in at AIIMS Delhi Laboratory**

**b. Eliminating the 3 Points for NfL and the 1 Point for pTau181 that are Clearly Off the Line**

**Figure S3.** **Distributions of Harmonized Neurodegenerative Biomarkers in India (N=2,151) and the US (N=3,466)**


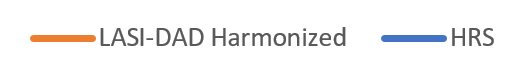


**Table S1. Harmonization Results for LASI-DAD Sample between AIIMS Delhi Laboratory and the University of Minnesota**

|  | N | Mean (SD) |
| --- | --- | --- |
| GFAP | 2673 | 147.74 (107.50) |
| Harmonized GFAP |  | 147.15 (110.04) |
| NfL | 2613 | 44.17 (80.43) |
| Harmonized NfL |  | 42.51 (74.65) |
| Aβ42 | 2182 | 3.27 (2.76) |
| Harmonized Aβ42 |  | 3.53 (2.42) |
| Aβ40 | 2558 | 49.16 (37.73) |
| Harmonized Aβ40 |  | 54.48 (32.97) |
| Aβ42/Aβ40 | 2175 | 0.07 (0.05) |
| Harmonized Aβ42/Aβ40 |  | 0.06 (0.04) |
| pTau181 | 2695 | 41.99 (31.45) |
| Harmonized pTau181 |  | 43.19 (29.37) |

**Table S2. CFA Factor Loadings and Fit Statistics for Neurodegenerative Biomarker Factors in LASI-DAD and HRS**

|  | LASI-DAD | HRS |
| --- | --- | --- |
| Standardized Factor Loadings |  |  |
| GFAP | 0.565 | 0.683 |
| NfL | 0.487 | 0.687 |
| pTau181 | 0.204 | 0.405 |
| Fit Statistics |  |  |
| CFI | 1.000 | 1.000 |
| TLI | 1.000 | 1.000 |
| RMSEA | 0.000 (90% CI: 0.000, 0.000) | 0.000 (90% CI: 0.000, 0.000) |
| SRMR | 0.000 | 0.000 |

CFI: Comparative Fit Index

TLI: Tucker-Lewis Index

RMEAS (Root Mean Square Error of Approximation)

SRMR (Standardized Root Mean Square Residual)

**Table S3. Coefficients for Figure 4**

|  | **LASI-DAD (N=2151)** | | | | | | | | | | | |
| --- | --- | --- | --- | --- | --- | --- | --- | --- | --- | --- | --- | --- |
|  | Individual Models | | | | Combined Model | | | | Biomarker Factor Score | | | |
|  | b | *P* | lower CI | upper CI | b | *P* | lower CI | upper CI | b | *P* | lower CI | upper CI |
| zGFAP | -0.10 | < 0.01 | -0.14 | -0.06 | -0.07 | < 0.01 | -0.11 | -0.02 |  |  |  |  |
| zNfL | -0.09 | < 0.01 | -0.13 | -0.06 | -0.07 | < 0.01 | -0.12 | -0.03 |  |  |  |  |
| zAβ42/Aβ40 | 0.04 | 0.03 | 0.00 | 0.08 | 0.04 | 0.08 | -0.00 | 0.08 |  |  |  |  |
| zpTau181 | 0.01 | 0.80 | -0.03 | 0.04 | 0.03 | 0.15 | -0.01 | 0.07 |  |  |  |  |
| zBiomarker Factor Score |  |  |  |  |  |  |  |  | -0.11 | < 0.01 | -0.15 | -0.07 |
| Adj R^2^ | zGFAP  0.18 | zNfL  0.18 | zAβ42/Aβ40  0.17 | zpTau181  0.17 | 0.19 | | | | 0.18 | | | |
|  |  |  |  |  |  |  |  |  |  |  |  |  |
|  | **HRS (N=2323)** | | | | | | | | | | | |
|  | Individual Models | | | | Combined Model | | | | Biomarker Factor Score | | | |
|  | b | *P* | lower CI | upper CI | b | *P* | lower CI | upper CI | b | *P* | lower CI | upper CI |
| zGFAP | -0.20 | < 0.01 | -0.24 | -0.16 | -0.16 | < 0.01 | -0.20 | -0.11 |  |  |  |  |
| zNfL | -0.15 | < 0.01 | -0.19 | -0.12 | -0.10 | < 0.01 | -0.14 | -0.06 |  |  |  |  |
| zAβ42/Aβ40 | -0.04 | < 0.01 | -0.08 | -0.01 | -0.04 | 0.01 | -0.07 | -0.01 |  |  |  |  |
| zpTau181 | -0.09 | < 0.01 | -0.12 | -0.05 | -0.04 | 0.04 | -0.08 | -0.00 |  |  |  |  |
| zBiomarker Factor Score |  |  |  |  |  |  |  |  | -0.23 | < 0.01 | -0.27 | -0.19 |
| Adj R^2^ | zGFAP  0.35 | zNfL  0.34 | zAβ42/Aβ40  0.33 | zpTau181  0.33 | 0.36 | | | | 0.36 | | | |

**Table S4. Odds Ratios for Figure 5**

|  | **LASI-DAD (N=1405)** | | | | | | | | | | | |
| --- | --- | --- | --- | --- | --- | --- | --- | --- | --- | --- | --- | --- |
|  | Individual Models | | | | Combined Model | | | | Biomarker Factor Score | | | |
|  | OR | *P* | lower CI | upper CI | OR | *P* | lower CI | upper CI | OR | *P* | lower CI | upper CI |
| zGFAP | 1.45 | < 0.01 | 1.21 | 1.74 | 1.34 | < 0.01 | 1.10 | 1.62 |  |  |  |  |
| zNfL | 1.29 | < 0.01 | 1.12 | 1.49 | 1.21 | 0.02 | 1.04 | 1.41 |  |  |  |  |
| zAβ42/Aβ40 | 0.79 | 0.18 | 0.57 | 1.11 | 0.84 | 0.32 | 0.60 | 1.19 |  |  |  |  |
| zpTau181 | 1.10 | 0.24 | 0.94 | 1.30 | 1.03 | 0.81 | 0.81 | 1.31 |  |  |  |  |
| zBiomarker Factor Score |  |  |  |  |  |  |  |  | 1.53 | < 0.01 | 1.28 | 1.83 |
| Pseudo R^2^ | zGFAP  0.14 | zNfL  0.13 | zAβ42/Aβ40  0.11 | zpTau181  0.11 | 0.15 | | | | 0.15 | | | |
| -2Log L | 619.78 | 622.71 | 632.60 | 633.40 | 612.60 | | | | 613.86 | | | |

|  | **HRS (N=2333)** | | | | | | | | | | | |
| --- | --- | --- | --- | --- | --- | --- | --- | --- | --- | --- | --- | --- |
|  | Individual Models | | | | Combined Model | | | | Biomarker Factor Score | | | |
|  | OR | *P* | lower CI | upper CI | OR | *P* | lower CI | upper CI | OR | *P* | lower CI | upper CI |
| zGFAP | 1.67 | < 0.01 | 1.45 | 1.93 | 1.61 | < 0.01 | 1.38 | 1.87 |  |  |  |  |
| zNfL | 1.30 | < 0.01 | 1.14 | 1.48 | 1.17 | 0.01 | 1.04 | 1.32 |  |  |  |  |
| zAβ42/Aβ40 | 1.14 | 0.02 | 1.03 | 1.27 | 1.15 | 0.01 | 1.03 | 1.28 |  |  |  |  |
| zpTau181 | 1.10 | 0.14 | 0.97 | 1.25 | 0.98 | 0.80 | 0.81 | 1.17 |  |  |  |  |
| zBiomarker Factor Score |  |  |  |  |  |  |  |  | 1.62 | < 0.01 | 1.40 | 1.87 |
| Pseudo R^2^ | zGFAP  0.16 | zNfL  0.12 | zAβ42/Aβ40  0.11 | zpTau181  0.11 | 0.17 | | | | 0.15 | | | |
| -2Log L | 956.58 | 988.93 | 1001.17 | 1004.67 | 945.09 | | | | 962.57 | | | |

**Table S5. Odds Ratios for Figure 6**

|  | **LASI-DAD (N=1128)** | | | | | | | | | | | | | | | | | | |
| --- | --- | --- | --- | --- | --- | --- | --- | --- | --- | --- | --- | --- | --- | --- | --- | --- | --- | --- | --- |
|  | Cognitively Decline | | | | | | | | | | Died | | | | | | | | |
|  | OR | | *P* | | | lower CI | | upper CI | | | OR | | | *P* | | lower CI | | | upper CI |
| zGFAP | 1.06 | | 0.55 | | | 0.87 | | 1.30 | | | 1.48 | | | < 0.01 | | 1.18 | | | 1.85 |
| zNfL | 1.09 | | 0.69 | | | 0.72 | | 1.63 | | | 3.35 | | | < 0.01 | | 2.20 | | | 5.10 |
| zAβ42/Aβ40 | 0.93 | | 0.47 | | | 0.76 | | 1.14 | | | 0.76 | | | 0.04 | | 0.58 | | | 0.99 |
| zpTau181 | 0.98 | | 0.78 | | | 0.85 | | 1.14 | | | 1.00 | | | 0.96 | | 0.82 | | | 1.21 |
| zBiomarker Factor Score | 1.06 | | 0.62 | | | 0.85 | | 1.31 | | | 1.76 | | | < 0.01 | | 1.39 | | | 2.24 |
|  |  | |  | | |  | |  | | |  | | |  | |  | | |  |
| Pseudo R^2^ | zGFAP | | | zNfL | | | | | zAβ42/Aβ40 | | | zpTau181 | | | | | zBiomarker Factor Score | | |
|  | 0.11 | | | 0.17 | | | | | 0.10 | | | 0.09 | | | | | 0.13 | | |
| -2Log L | 2171.41 | | | 2096.83 | | | | | 2182.98 | | | 2187.40 | | | | | 2150.11 | | |
|  |  |  | | |  | |  | | |  | | |  | |  | | |  | |
|  | **HRS (2989)** | | | | | | | | | | | | | | | | | | |
|  | Cognitively Decline | | | | | | | | | | Died | | | | | | | | |
|  | OR | | *P* | | | lower CI | | upper CI | | | OR | | | *P* | | lower CI | | | upper CI |
| zGFAP | 1.19 | | < 0.01 | | | 1.05 | | 1.33 | | | 1.31 | | | < 0.01 | | 1.13 | | | 1.53 |
| zNfL | 1.13 | | 0.12 | | | 0.97 | | 1.33 | | | 1.72 | | | < 0.01 | | 1.43 | | | 2.06 |
| zAβ42/Aβ40 | 0.99 | | 0.75 | | | 0.91 | | 1.07 | | | 0.78 | | | < 0.01 | | 0.65 | | | 0.94 |
| zpTau181 | 1.04 | | 0.49 | | | 0.93 | | 1.17 | | | 1.32 | | | < 0.01 | | 1.15 | | | 1.52 |
| zBiomarker Factor Score | 1.19 | | 0.01 | | | 1.04 | | 1.36 | | | 1.71 | | | < 0.01 | | 1.46 | | | 2.01 |
|  |  | | | | |  | | | | |  | | | | |  | | | |
|  | zGFAP | | | zNfL | | | | | zAβ42/Aβ40 | | | zpTau181 | | | | | zBiomarker Factor Score | | |
| Pseudo R^2^ | 0.15 | | | 0.16 | | | | | 0.15 | | | 0.15 | | | | | 0.16 | | |
| -2Log L | 5353.00 | | | 5316.33 | | | | | 5358.23 | | | 5346.04 | | | | | 5317.78 | | |

**MODELS INCLUDING CONTROL FOR Caste and Rural Residency (LASI-DAD only) and Education**

Note for Tables S6 – S8: Runs were done for models with individual neurodegenerative biomarkers.

Differences in significant results between these results with additional controls of caste, rural residency and education, and those in the main body of the paper are highlighted in yellow.

**Table S6. Coefficients for the Results on Cognitive Function in Figure 4 and Table S3 (yellow indicates change in significance) when Caste, Rural Residency (LASI-DAD only) and Education are Controlled**

|  | **LASIDAD** | | | | **HRS** | | | |
| --- | --- | --- | --- | --- | --- | --- | --- | --- |
|  | b | *P* | lower CI | upper CI | b | *P* | lower CI | upper CI |
| zGFAP | -0.10 | < 0.01 | -0.13 | -0.06 | -0.19 | < 0.01 | -0.23 | -0.16 |
| zNfL | -0.09 | < 0.01 | -0.12 | -0.06 | -0.13 | < 0.01 | -0.17 | -0.10 |
| zAβ42/Aβ40 | 0.03 | 0.05 | -0.00 | 0.06 | -0.04 | 0.01 | -0.07 | -0.01 |
| zpTau181 | -0.01 | 0.40 | -0.04 | 0.02 | -0.09 | < 0.01 | -0.12 | -0.05 |
|  | zGFAP | zNfL | zAβ42/Aβ40 | zpTau181 | zGFAP | zNfL | zAβ42/Aβ40 | zpTau181 |
| Adj R^2^ | 0.53 | 0.52 | 0.52 | 0.52 | 0.46 | 0.45 | 0.44 | 0.44 |

**Table S7. ORs for the Results on Dementia in Figure 5 and Table S4 (yellow indicates change) when Caste, Rural Residency (LASI-DAD only) and Education are Controlled**

|  | **LASIDAD** | | | | **HRS** | | | |
| --- | --- | --- | --- | --- | --- | --- | --- | --- |
|  | OR | *P* | lower CI | upper CI | OR | *P* | lower CI | upper CI |
| zGFAP | 1.46 | < 0.01 | 1.21 | 1.77 | 1.68 | < 0.01 | 1.46 | 1.94 |
| zNfL | 1.28 | < 0.01 | 1.11 | 1.49 | 1.29 | < 0.01 | 1.13 | 1.47 |
| zAβ42/Aβ40 | 0.74 | 0.09 | 0.52 | 1.05 | 1.14 | 0.02 | 1.02 | 1.27 |
| zpTau181 | 1.16 | 0.10 | 0.97 | 1.38 | 1.10 | 0.16 | 0.96 | 1.25 |
|  | zGFAP | zNfL | zAβ42/Aβ40 | zpTau181 | zGFAP | zNfL | zAβ42/Aβ40 | zpTau181 |
| Pseudo R^2^ | 0.18 | 0.17 | 0.16 | 0.15 | 0.16 | 0.13 | 0.11 | 0.11 |
| -2Log L | 679.52 | 679.52 | 679.52 | 679.52 | 1101.11 | 1101.11 | 1101.11 | 1101.11 |

**Table S8. ORs for the Results in Figure 6 and Table S5 (yellow indicates change) when Caste, Rural Residency (LASI-DAD only) and Education are Controlled**

|  | **LASIDAD** | | | | | | | |
| --- | --- | --- | --- | --- | --- | --- | --- | --- |
|  | Cognitive Decline | | | | Died | | | |
|  | OR | *P* | lower CI | upper CI | OR | *P* | lower CI | upper CI |
| zGFAP | 1.14 | 0.74 | 0.84 | 1.27 | 1.41 | < 0.01 | 1.12 | 1.78 |
| zNfL | 1.04 | 0.86 | 0.69 | 1.57 | 3.08 | < 0.01 | 2.01 | 4.71 |
| zAβ42/Aβ40 | 0.92 | 0.46 | 0.75 | 1.14 | 0.73 | 0.02 | 0.56 | 0.96 |
| zpTau181 | 0.95 | 0.53 | 0.82 | 1.11 | 0.97 | 0.75 | 0.79 | 1.18 |
|  | zGFAP | | zNfL | | zAβ42/Aβ40 | | zpTau181 | |
| Pseudo R^2^ | 0.14 | | 0.20 | | 0.14 | | 0.13 | |
| -2Log L | 2272.48 | | 2272.48 | | 2272.48 | | 2272.48 | |

|  | **HRS** | | | | | | | | | | |
| --- | --- | --- | --- | --- | --- | --- | --- | --- | --- | --- | --- |
|  | Cognitive Decline | | | | | | Died | | | | |
|  | OR | *P* | lower CI | | upper CI | | OR | *P* | lower CI | | upper CI |
| zGFAP | 1.18 | 0.01 | 1.05 | | 1.34 | | 1.33 | < 0.01 | 1.14 | | 1.56 |
| zNfL | 1.12 | 0.16 | 0.96 | | 1.31 | | 1.69 | < 0.01 | 1.41 | | 2.03 |
| zAβ42/Aβ40 | 0.99 | 0.74 | 0.91 | | 1.07 | | 0.76 | < 0.01 | 0.63 | | 0.92 |
| zpTau181 | 1.04 | 0.50 | 0.93 | | 1.17 | | 1.35 | < 0.01 | 1.18 | | 1.55 |
|  | zGFAP | | | zNfL | | zAβ42/Aβ40 | | | | zpTau181 | |
| Pseudo R^2^ | 0.16 | | | 0.17 | | 0.16 | | | | 0.17 | |
| -2Log L | 5755.36 | | | 5755.36 | | 5755.36 | | | | 5755.36 | |

**Table S9. ORs for the Results in Figure 6 based on Transformed HRS Scale: HRS (N=2989)**

|  | **Original LW Scores** | | | | | | | | | | | | | | |  |
| --- | --- | --- | --- | --- | --- | --- | --- | --- | --- | --- | --- | --- | --- | --- | --- | --- |
|  | Cognitively Decline | | | | | | | | | Died | | | | | |  |
|  | OR | | *P* | | | lower CI | | upper CI | | OR | | *P* | | lower CI | upper CI |  |
| zGFAP | 1.10 | | 0.05 | | | 1.00 | | 1.23 | | 1.24 | | < 0.01 | | 1.08 | 1.43 |  |
| zNfL | 1.20 | | 0.02 | | | 1.04 | | 1.40 | | 1.77 | | < 0.01 | | 1.49 | 2.11 |  |
| zAβ42/Aβ40 | 1.03 | | 0.44 | | | 0.95 | | 1.12 | | 0.80 | | 0.02 | | 0.67 | 0.96 |  |
| zpTau181 | 1.05 | | 0.42 | | | 0.94 | | 1.17 | | 1.32 | | < 0.01 | | 1.16 | 1.51 |  |
| zBiomarker Factor Score | 1.17 | | 0.01 | | | 1.03 | | 1.32 | | 1.66 | | < 0.01 | | 1.43 | 1.93 |  |
|  |  |  | |  |  | |  | |  | |  | |  | | | |
|  | **Transformed LW Scores** | | | | | | | | | | | | | | |  |
|  | Cognitively Decline | | | | | | | | | Died | | | | | |  |
|  | OR | | *P* | | | lower CI | | upper CI | | OR | | *P* | | lower CI | upper CI |  |
| zGFAP | 1.18 | | 0.01 | | | 1.05 | | 1.32 | | 1.31 | | < 0.01 | | 1.12 | 1.52 |  |
| zNfL | 1.13 | | 0.14 | | | 0.96 | | 1.32 | | 1.71 | | < 0.01 | | 1.43 | 2.05 |  |
| zAβ42/Aβ40 | 0.99 | | 0.77 | | | 0.91 | | 1.07 | | 0.78 | | 0.01 | | 0.65 | 0.94 |  |
| zpTau181 | 1.04 | | 0.50 | | | 0.93 | | 1.17 | | 1.32 | | < 0.01 | | 1.15 | 1.52 |  |
| zBiomarker Factor Score | 1.18 | | 0.01 | | | 1.04 | | 1.35 | | 1.70 | | < 0.01 | | 1.45 | 2.00 |  |

Note: Each biomarker was included in the model separately.

**MODELS INCLUDING CONTROL FOR PLATE**

Note for Tables S10 – S12: Runs were done for models with individual neurodegenerative biomarkers as multiple markers would require multiple plate controls. For Aβ42/Aβ40, while most were done on the same plate, 4 were done on different plates and the Aβ40 plate was used as control.

Differences in significant results between these results with plate controls and those in the main body of the paper are highlighted in yellow.

**Table S10. Coefficients for the Results on Cognitive Function in Figure 4 and Table S2 (yellow indicates change in significance) when Plates is Controlled**

|  | **LASIDAD** | | | | **HRS** | | | |
| --- | --- | --- | --- | --- | --- | --- | --- | --- |
|  | b | *P* | lower CI | upper CI | b | *P* | lower CI | upper CI |
| zGFAP | -0.10 | < 0.01 | -0.14 | -0.06 | -0.20 | < 0.01 | -0.23 | -0.16 |
| zNfL | -0.10 | < 0.01 | -0.13 | -0.06 | -0.13 | < 0.01 | -0.16 | -0.10 |
| zAβ42/Aβ40 | 0.04 | 0.06 | -0.00 | 0.08 | -0.03 | 0.06 | -0.06 | 0.00 |
| zpTau181 | 0.01 | 0.77 | -0.04 | 0.05 | -0.09 | < 0.01 | -0.12 | -0.05 |
|  | zGFAP | zNfL | zAβ42/Aβ40 | zpTau181 | zGFAP | zNfL | zAβ42/Aβ40 | zpTau181 |
| Adj R^2^ | 0.20 | 0.20 | 0.19 | 0.19 | 0.47 | 0.45 | 0.44 | 0.45 |

**Table S11. ORs for the Results on Dementia in Figure 5 and Table S3 (yellow indicates change) when Plate is Controlled**

|  | **LASIDAD** | | | | **HRS** | | | |
| --- | --- | --- | --- | --- | --- | --- | --- | --- |
|  | OR | *P* | lower CI | upper CI | OR | *P* | lower CI | upper CI |
| zGFAP | 1.64 | < 0.01 | 1.32 | 2.05 | 1.45 | < 0.01 | 1.25 | 1.68 |
| zNfL | 1.38 | < 0.01 | 1.18 | 1.63 | 1.14 | 0.03 | 1.01 | 1.29 |
| zAβ42/Aβ40 | 0.75 | 0.15 | 0.51 | 1.10 | 1.07 | 0.91 | 0.93 | 1.22 |
| zpTau181 | 1.43 | 0.02 | 1.05 | 1.95 | 1.12 | 0.11 | 0.98 | 1.28 |
|  | zGFAP | zNfL | zAβ42/Aβ40 | zpTau181 | zGFAP | zNfL | zAβ42/Aβ40 | zpTau181 |
| Pseudo R^2^ | 0.24 | 0.24 | 0.21 | 0.23 | 0.24 | 0.23 | 0.23 | 0.23 |
| -2Log L | 696.27 | 696.27 | 696.27 | 696.27 | 1087.91 | 1087.91 | 1087.91 | 1087.91 |

**Table S12. ORs for the Results in Figure 6 and Table S4 (yellow indicates change) when Plate is Controlled**

|  | **LASIDAD** | | | | | | | |
| --- | --- | --- | --- | --- | --- | --- | --- | --- |
|  | Cognitive Decline | | | | Died | | | |
|  | OR | *P* | lower CI | upper CI | OR | *P* | lower CI | upper CI |
| zGFAP | 1.12 | 0.35 | 0.89 | 1.40 | 1.53 | < 0.01 | 1.19 | 1.98 |
| zNfL | 1.11 | 0.63 | 0.72 | 1.73 | 3.67 | < 0.01 | 2.31 | 5.82 |
| zAβ42/Aβ40 | 1.01 | 0.95 | 0.79 | 1.28 | 0.77 | 0.10 | 0.56 | 1.05 |
| zpTau181 | 0.96 | 0.58 | 0.81 | 1.12 | 1.00 | 0.99 | 0.82 | 1.21 |
|  | zGFAP | | zNfL | | zAβ42/Aβ40 | | zpTau181 | |
| Pseudo R^2^ | 0.25 | | 0.31 | | 0.24 | | 0.23 | |
| -2Log L | 2281.37 | | 2281.37 | | 2281.37 | | 2281.37 | |

|  | **HRS** | | | | | | | | | | |
| --- | --- | --- | --- | --- | --- | --- | --- | --- | --- | --- | --- |
|  | Cognitive Decline | | | | | | Died | | | | |
|  | OR | *P* | lower CI | | upper CI | | OR | *P* | lower CI | | upper CI |
| zGFAP | 1.15 | 0.02 | 1.02 | | 1.30 | | 1.30 | < 0.01 | 1.11 | | 1.52 |
| zNfL | 1.11 | 0.21 | 0.94 | | 1.30 | | 1.74 | < 0.01 | 1.44 | | 2.10 |
| zAβ42/Aβ40 | 0.99 | 0.74 | 0.91 | | 1.07 | | 0.81 | 0.02 | 0.67 | | 0.97 |
| zpTau181 | 1.04 | 0.50 | 0.93 | | 1.17 | | 1.32 | < 0.01 | 1.15 | | 1.51 |
|  | zGFAP | | | zNfL | | zAβ42/Aβ40 | | | | zpTau181 | |
| Pseudo R^2^ | 0.18 | | | 0.19 | | 0.18 | | | | 0.18 | |
| -2Log L | 5760.47 | | | 5760.47 | | 5760.47 | | | | 5760.47 | |
