# Supplementary material for: Association of blood-based neurodegenerative biomarkers with cognitive functioning and dementia in India (LASI-DAD) and the United States (HRS)
Source: Am J Epidemiol. Author manuscript; Available in PMC 2025 Oct 7. (PMC12497661; doi:10.1093/aje/kwaf179)
Supplement: Supplementary file 1 [file NIHMS2114315-supplement-supplementary_material.docx]

Supplemental Material

Association of Blood-based Neurodegenerative Biomarkers with Cognitive Functioning and Dementia in India (LASI-DAD) and the United States (HRS)

Jung Ki Kim, Masroor Anwar, Abhishek Gupta, Bharat Thyagarajan, Peifeng Hu, Jessica D. Faul, David R. Weir, Kenneth M. Langa, Jinkook Lee, Sharmistha Dey, Eileen M. Crimmins

Figure S1. Sample Chart

Appendix S1. Harmonization of the Neurodegenerative Assays across Two Labs

Figure S2. a. Assay Validation in at AIIMS Delhi Laboratory

Figure S2. b. Eliminating the 3 Points for NfL and the 1 Point for pTau181 that are Clearly Off the Line

Figure S3. Distributions of Harmonized Neurodegenerative Biomarkers in India (N=2,151) and the US (N=3,466)

Table S1. CFA Factor Loadings and Fit Statistics for Neurodegenerative Biomarker Factors in LASI-DAD and HRS

Table S2. Harmonization Results for LASI-DAD Sample between AIIMS Delhi Laboratory and the University of Minnesota

Table S3. Coefficients for Figure 4

Table S4. Odds Ratios for Figure 5

Table S5. Odds Ratios for Figure 6

Table S6. Coefficients for the Results on Cognitive Function in Figure 4 and Table S2 (yellow indicates change in significance) when Caste, Rural Residency (LASI-DAD only) and Education are Controlled

Table S7. ORs for the Results on Dementia in Figure 5 and Table S3 (yellow indicates change) when Caste, Rural Residency (LASI-DAD only) and Education are Controlled

Table S8. ORs for the Results in Figure 6 and Table S4 (yellow indicates change) when Caste, Rural Residency (LASI-DAD only) and Education are Controlled

Table S9. ORs for the Results in Figure 6 based on Transformed HRS Scale for HRS

Table S10. Coefficients for the Results on Cognitive Function in Figure 4 and Table S2 (yellow indicates change in significance) when Plates is Controlled

Table S11. ORs for the Results on Dementia in Figure 5 and Table S3 (yellow indicates change) when Plate is Controlled

Table S12. ORs for the Results in Figure 6 and Table S4 (yellow indicates change) when Plate is Controlled

Figure S1. Sample Chart

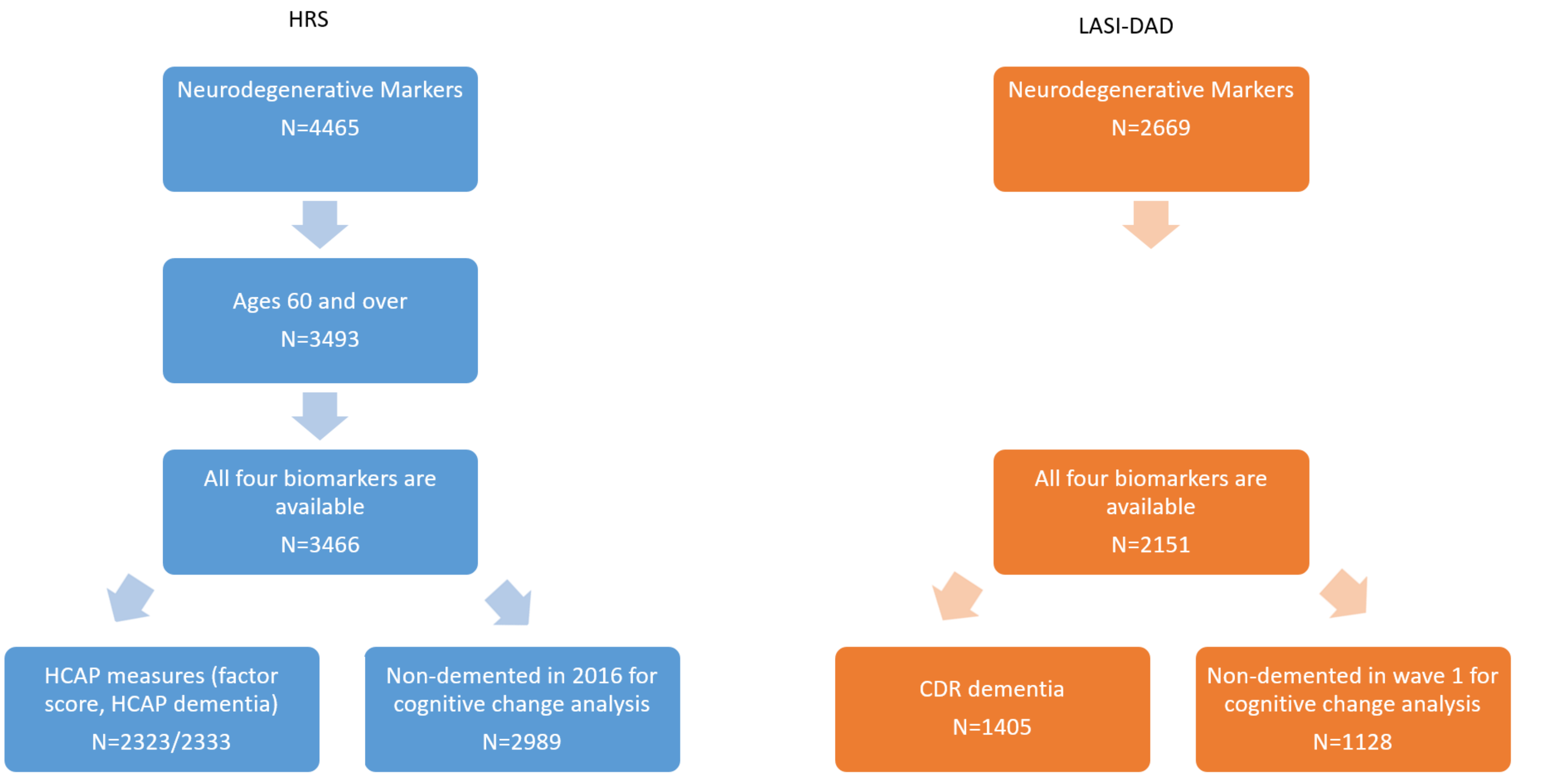

Appendix S1. Harmonization of the Neurodegenerative Assays across Two Labs

Neurodegenerative markers for the 2016 HRS sample were assayed in the Advanced Research and Diagnostic Laboratory (ARDL) in the Department of Laboratory Medicine and Pathology at the University of Minnesota in 2022 and 2023. These markers were done after a pilot test to determine the validity and reliability of neurodegenerative assays collected in the field under the HRS protocol and shipped overnight to the laboratory. The details and results of this initial pilot are provided in Panikkar et al. 2023.[25]

In order to ensure comparability across countries for the neurodegenerative markers, the LASI-DAD lab members were trained on the instrument used by HRS at the UMN. The same Quanterix HDX Simoa analyzer, a highly sensitive immunoassay platform, was purchased for use at AIIMS Delhi laboratory and a comparison of assay results was conducted from duplicate plasma specimens prepared and assayed in Minnesota and in Delhi before beginning the analysis of study samples.

The harmonization of assay preparation for HRS and LASI-DAD was accomplished through training of AIIMS laboratory staff from Delhi at the University of Minnesota laboratory and purchase of an identical machine for use at AIIMS, a Quanterix HDX fully-automated SIMOA analyser. After training, a total of 81 duplicate plasma samples were plated at Minnesota and sent via a cold chain to AIIMS. These were then assayed for Aβ42, Aβ40, NfL, GFAP, t-tau and pTau181 at the two labs to compare results. The laboratories were blinded to the results to avoid bias during the analysis.

The results of the assays from the two labs are shown below in Figure S2a and b. There is a very similar ranking of the assay values from the two labs with correlation coefficients ranging from 0.88 for NfL to 0.98 for Aβ40 (Figure S2a). NfL and pTau181 have relatively low R2. In order to get a better equation for harmonization, we recompute the equation eliminating the 3 points for NfL that are clearly off the line and the 1 point for pTau181 that is clearly off the line. We assumed that these off-diagonal points represented random technical errors either at

UMN or at the AIIMS laboratory and so these data points would not contribute meaningfully to the comparison of assays across the two laboratories. When 3 cases that are obviously out of line are eliminated from NfL (Figure S2b), the correlation is .98; for pTau181the correlation increases to 0.89. This makes it clear that the labs are producing similar assay results and can be compared. We should note that there is some difference in level which results in our using z scored measures in the analysis. It is also possible to use the equations in Figure S2 to create harmonized values for the two labs. We do this by inserting the assayed value for LASI-DAD into the equations and estimate the value for LASI-DAD in University of Minnesota values. The effect of doing this is shown in Table S2 which compares the original means and the harmonized means for LASI-DAD and in Figure S3 which compares the distribution of harmonized values for LASI-DAD and HRS.

Panikkar D, Vivek S, Crimmins E, et al. Pre-analytical variables influencing stability of blood-based biomarkers of neuropathology. *J Alzheimers Dis*. 2023;**95**(2):735-748. https://doi.org/10.3233/JAD-230384.

Figure S2.

a. Assay Validation in at AIIMS Delhi Laboratory

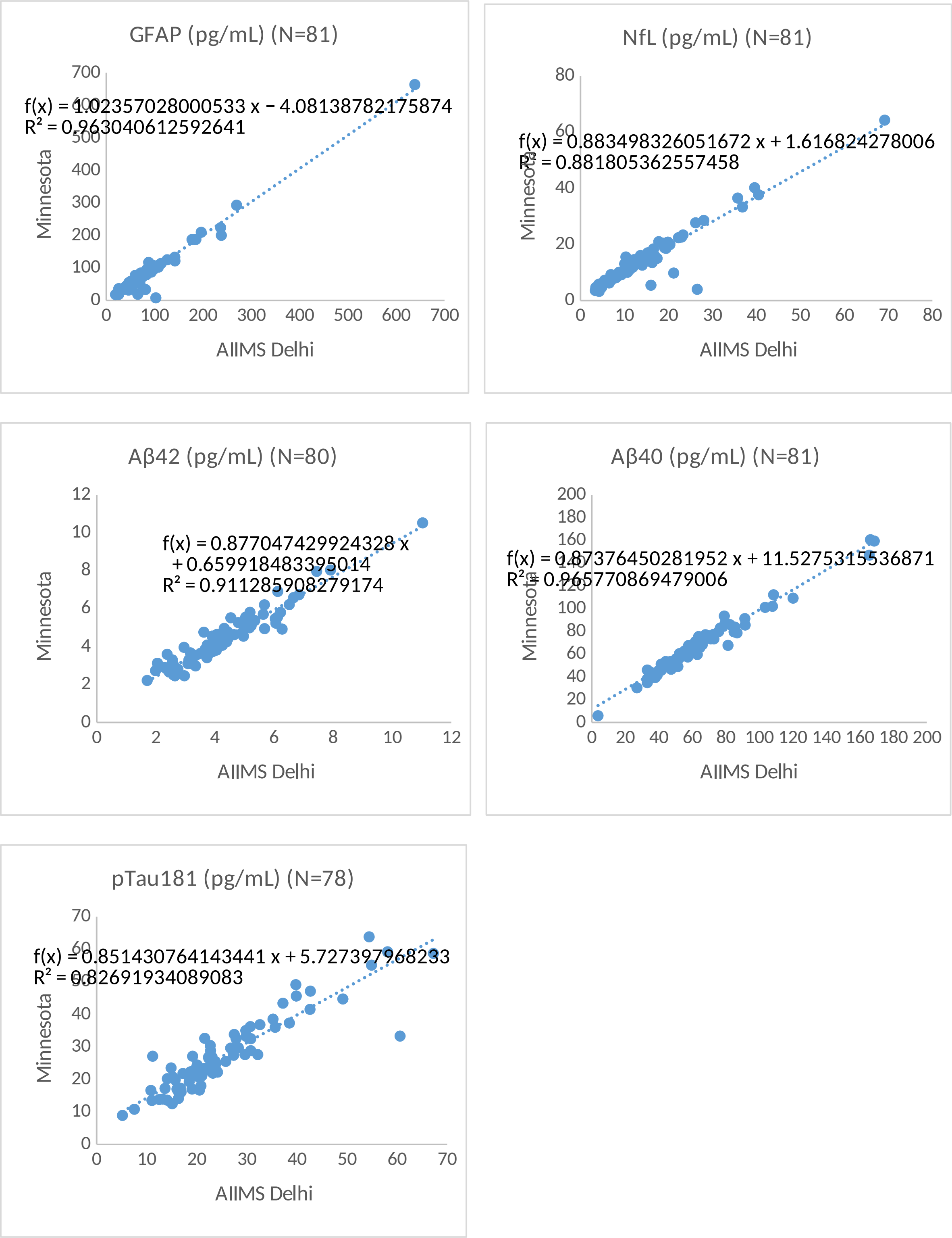

b. Eliminating the 3 Points for NfL and the 1 Point for pTau181 that are Clearly Off the Line

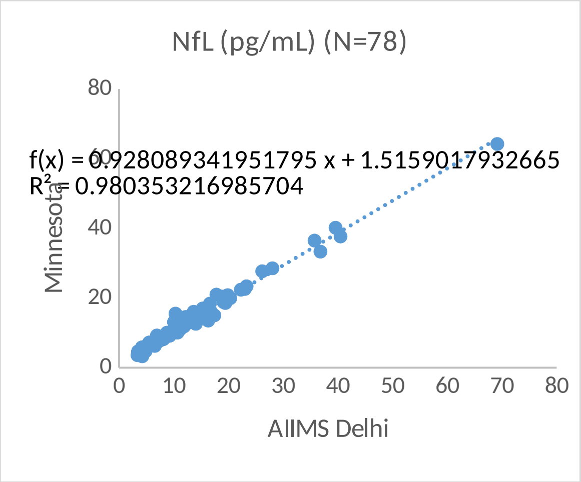

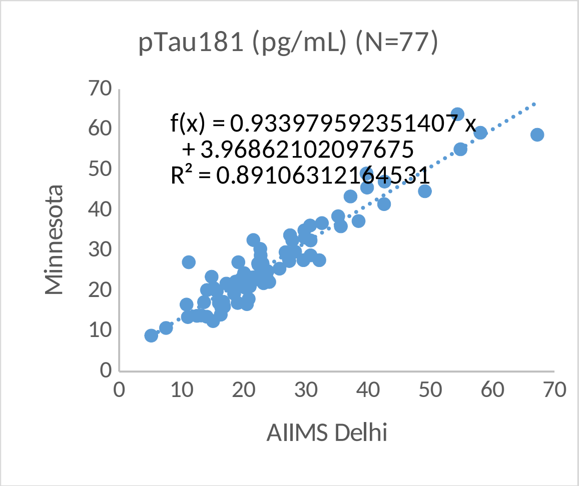

Figure S3. **Distributions of Harmonized Neurodegenerative Biomarkers in India (N=2,151) and the US (N=3,466)**

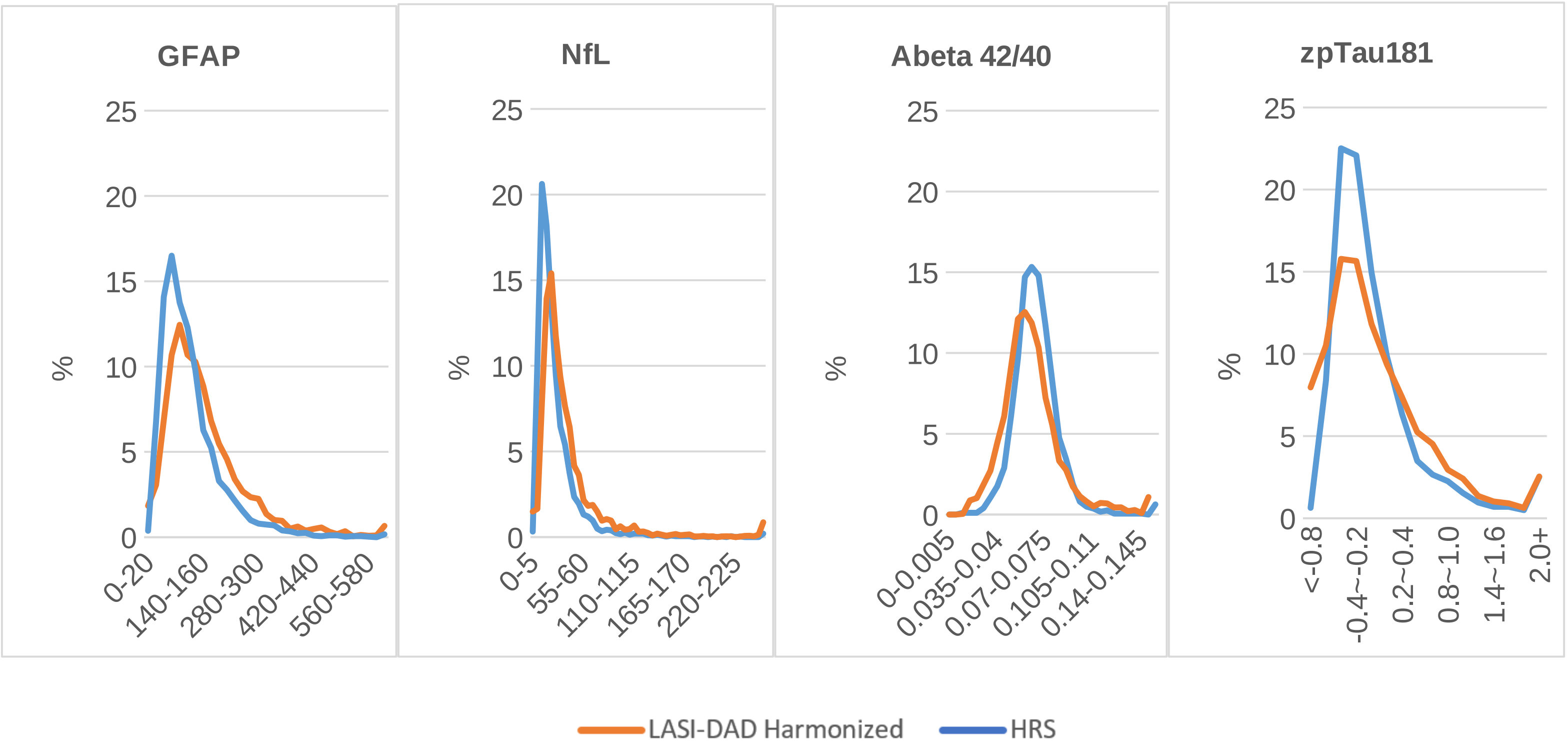

Table S1. CFA Factor Loadings and Fit Statistics for Neurodegenerative Biomarker Factors in LASI-DAD and HRS

	LASI-DAD	HRS
Standardized Factor Loadings		
GFAP	0.565	0.683
NfL	0.487	0.687
pTau181	0.204	0.405
Fit Statistics		
CFI	1.000	1.000
TLI	1.000	1.000
RMSEA	0.000 (90% CI: 0.000, 0.000)	0.000 (90% CI: 0.000, 0.000)
SRMR	0.000	0.000

CFI: Comparative Fit Index
TLI: Tucker-Lewis Index
RMEAS (Root Mean Square Error of Approximation)
SRMR (Standardized Root Mean Square Residual)

Table S2. Harmonization Results for LASI-DAD Sample between AIIMS Delhi Laboratory and the University of Minnesota

	N	Mean (SD)
GFAP	2673	147.74 (107.50)
Harmonized GFAP		147.15 (110.04)
NfL	2613	44.17 (80.43)
Harmonized NfL		42.51 (74.65)
Aβ42	2182	3.27 (2.76)
Harmonized Aβ42		3.53 (2.42)
Aβ40	2558	49.16 (37.73)
Harmonized Aβ40		54.48 (32.97)
Aβ42/Aβ40	2175	0.07 (0.05)
Harmonized Aβ42/Aβ40		0.06 (0.04)
pTau181	2695	41.99 (31.45)
Harmonized pTau181		43.19 (29.37)

Table S3. Coefficients for Figure 4

	**LASI-DAD (N=2151)**											
	Individual Models				Combined Model				Biomarker Factor Score			
	b	*P*	lower CI	upper CI	b	*P*	lower CI	upper CI	b	*P*	lower CI	upper CI
zGFAP	-0.10	< 0.01	-0.14	-0.06	-0.07	< 0.01	-0.11	-0.02				
zNfL	-0.09	< 0.01	-0.13	-0.06	-0.07	< 0.01	-0.12	-0.03				
zAβ42/Aβ40	0.04	0.03	0.00	0.08	0.04	0.08	-0.00	0.08				
zpTau181	0.01	0.80	-0.03	0.04	0.03	0.15	-0.01	0.07				
zBiomarker Factor Score									-0.11	< 0.01	-0.15	-0.07
Adj R^2	zGFAP 0.18	zNfL 0.18	zAβ42/Aβ40 0.17	zpTau181 0.17	0.19				0.18			

	**HRS (N=2323)**											
	Individual Models				Combined Model				Biomarker Factor Score			
	b	*P*	lower CI	upper CI	b	*P*	lower CI	upper CI	b	*P*	lower CI	upper CI
zGFAP	-0.20	< 0.01	-0.24	-0.16	-0.16	< 0.01	-0.20	-0.11				
zNfL	-0.15	< 0.01	-0.19	-0.12	-0.10	< 0.01	-0.14	-0.06				
zAβ42/Aβ40	-0.04	< 0.01	-0.08	-0.01	-0.04	0.01	-0.07	-0.01				
zpTau181	-0.09	< 0.01	-0.12	-0.05	-0.04	0.04	-0.08	-0.00				
zBiomarker Factor Score									-0.23	< 0.01	-0.27	-0.19
Adj R^2	zGFAP 0.35	zNfL 0.34	zAβ42/Aβ40 0.33	zpTau181 0.33	0.36				0.36			

Table S4. Odds Ratios for Figure 5

	LASI-DAD (N=1405)											
	Individual Models				Combined Model				Biomarker Factor Score			
	OR	*P*	lower CI	upper CI	OR	*P*	lower CI	upper CI	OR	*P*	lower CI	upper CI
zGFAP	1.45	< 0.01	1.21	1.74	1.34	< 0.01	1.10	1.62				
zNfL	1.29	< 0.01	1.12	1.49	1.21	0.02	1.04	1.41				
zAβ42/Aβ40	0.79	0.18	0.57	1.11	0.84	0.32	0.60	1.19				
zpTau181	1.10	0.24	0.94	1.30	1.03	0.81	0.81	1.31				
zBiomarker Factor Score									1.53	< 0.01	1.28	1.83
Pseudo R^2	zGFAP 0.14	zNfL 0.13	zAβ42/Aβ40 0.11	zpTau181 0.11	0.15				0.15			
-2Log L	619.78	622.71	632.60	633.40	612.60				613.86			

	HRS (N=2333)											
	Individual Models				Combined Model				Biomarker Factor Score			
	OR	*P*	lower CI	upper CI	OR	*P*	lower CI	upper CI	OR	*P*	lower CI	upper CI
zGFAP	1.67	< 0.01	1.45	1.93	1.61	< 0.01	1.38	1.87				
zNfL	1.30	< 0.01	1.14	1.48	1.17	0.01	1.04	1.32				
zAβ42/Aβ40	1.14	0.02	1.03	1.27	1.15	0.01	1.03	1.28				
zpTau181	1.10	0.14	0.97	1.25	0.98	0.80	0.81	1.17				
zBiomarker Factor Score									1.62	< 0.01	1.40	1.87
Pseudo R^2	zGFAP 0.16	zNfL 0.12	zAβ42/Aβ40 0.11	zpTau181 0.11	0.17				0.15			
-2Log L	956.58	988.93	1001.17	1004.67	945.09				962.57			

Table S5. Odds Ratios for Figure 6

	LASI-DAD (N=1128)							
	Cognitively Decline				Died			
	OR	*P*	lower CI	upper CI	OR	*P*	lower CI	upper CI
zGFAP	1.06	0.55	0.87	1.30	1.48	< 0.01	1.18	1.85
zNfL	1.09	0.69	0.72	1.63	3.35	< 0.01	2.20	5.10
zAβ42/Aβ40	0.93	0.47	0.76	1.14	0.76	0.04	0.58	0.99
zpTau181	0.98	0.78	0.85	1.14	1.00	0.96	0.82	1.21
zBiomarker Factor Score	1.06	0.62	0.85	1.31	1.76	< 0.01	1.39	2.24

	zGFAP	zNfL	zAβ42/Aβ40	zpTau181	zBiomarker Factor Score
Pseudo R^2	0.11	0.17	0.10	0.09	0.13
-2Log L	2171.41	2096.83	2182.98	2187.40	2150.11

	HRS (2989)							
	Cognitively Decline				Died			
	OR	*P*	lower CI	upper CI	OR	*P*	lower CI	upper CI
zGFAP	1.19	< 0.01	1.05	1.33	1.31	< 0.01	1.13	1.53
zNfL	1.13	0.12	0.97	1.33	1.72	< 0.01	1.43	2.06
zAβ42/Aβ40	0.99	0.75	0.91	1.07	0.78	< 0.01	0.65	0.94
zpTau181	1.04	0.49	0.93	1.17	1.32	< 0.01	1.15	1.52
zBiomarker Factor Score	1.19	0.01	1.04	1.36	1.71	< 0.01	1.46	2.01

	zGFAP	zNfL	zAβ42/Aβ40	zpTau181	zBiomarker Factor Score
Pseudo R^2	0.15	0.16	0.15	0.15	0.16
-2Log L	5353.00	5316.33	5358.23	5346.04	5317.78

MODELS INCLUDING CONTROL FOR Caste and Rural Residency (LASI-DAD only) and Education

Note for Tables S6 – S8: Runs were done for models with individual neurodegenerative biomarkers.
Differences in significant results between these results with additional controls of caste, rural residency and education, and those in the main body of the paper are highlighted in yellow.

Table S6. Coefficients for the Results on Cognitive Function in Figure 4 and Table S3 (yellow indicates change in significance) when Caste, Rural Residency (LASI-DAD only) and Education are Controlled

	**LASIDAD**				**HRS**			
	b	*P*	lower CI	upper CI	b	*P*	lower CI	upper CI
zGFAP	-0.10	< 0.01	-0.13	-0.06	-0.19	< 0.01	-0.23	-0.16
zNfL	-0.09	< 0.01	-0.12	-0.06	-0.13	< 0.01	-0.17	-0.10
zAβ42/Aβ40	0.03	0.05	-0.00	0.06	-0.04	0.01	-0.07	-0.01
zpTau181	-0.01	0.40	-0.04	0.02	-0.09	< 0.01	-0.12	-0.05
	zGFAP	zNfL	zAβ42/Aβ40	zpTau181	zGFAP	zNfL	zAβ42/Aβ40	zpTau181
Adj R^2	0.53	0.52	0.52	0.52	0.46	0.45	0.44	0.44

Table S7. ORs for the Results on Dementia in Figure 5 and Table S4 (yellow indicates change) when Caste, Rural Residency (LASI-DAD only) and Education are Controlled

	**LASIDAD**				**HRS**			
	OR	*P*	lower CI	upper CI	OR	*P*	lower CI	upper CI
zGFAP	1.46	< 0.01	1.21	1.77	1.68	< 0.01	1.46	1.94
zNfL	1.28	< 0.01	1.11	1.49	1.29	< 0.01	1.13	1.47
zAβ42/Aβ40	0.74	0.09	0.52	1.05	1.14	0.02	1.02	1.27
zpTau181	1.16	0.10	0.97	1.38	1.10	0.16	0.96	1.25
	zGFAP	zNfL	zAβ42/Aβ40	zpTau181	zGFAP	zNfL	zAβ42/Aβ40	zpTau181
Pseudo R^2	0.18	0.17	0.16	0.15	0.16	0.13	0.11	0.11
-2Log L	679.52	679.52	679.52	679.52	1101.11	1101.11	1101.11	1101.11

Table S8. ORs for the Results in Figure 6 and Table S5 (yellow indicates change) when Caste, Rural Residency (LASI-DAD only) and Education are Controlled

	**LASIDAD**							
	Cognitive Decline				Died			
	OR	*P*	lower CI	upper CI	OR	*P*	lower CI	upper CI
zGFAP	1.14	0.74	0.84	1.27	1.41	< 0.01	1.12	1.78
zNfL	1.04	0.86	0.69	1.57	3.08	< 0.01	2.01	4.71
zAβ42/Aβ40	0.92	0.46	0.75	1.14	0.73	0.02	0.56	0.96
zpTau181	0.95	0.53	0.82	1.11	0.97	0.75	0.79	1.18
	zGFAP		zNfL		zAβ42/Aβ40		zpTau181	
Pseudo R^2	0.14		0.20		0.14		0.13	
-2Log L	2272.48		2272.48		2272.48		2272.48	

	**HRS**							
	Cognitive Decline				Died			
	OR	*P*	lower CI	upper CI	OR	*P*	lower CI	upper CI
zGFAP	1.18	0.01	1.05	1.34	1.33	< 0.01	1.14	1.56
zNfL	1.12	0.16	0.96	1.31	1.69	< 0.01	1.41	2.03
zAβ42/Aβ40	0.99	0.74	0.91	1.07	0.76	< 0.01	0.63	0.92
zpTau181	1.04	0.50	0.93	1.17	1.35	< 0.01	1.18	1.55
	zGFAP		zNfL		zAβ42/Aβ40		zpTau181	
Pseudo R^2	0.16		0.17		0.16		0.17	
-2Log L	5755.36		5755.36		5755.36		5755.36	

Table S9. ORs for the Results in Figure 6 based on Transformed HRS Scale: HRS (N=2989)

	**Original LW Scores**							
	Cognitively Decline				Died			
	OR	*P*	lower CI	upper CI	OR	*P*	lower CI	upper CI
zGFAP	1.10	0.05	1.00	1.23	1.24	< 0.01	1.08	1.43
zNfL	1.20	0.02	1.04	1.40	1.77	< 0.01	1.49	2.11
zAβ42/Aβ40	1.03	0.44	0.95	1.12	0.80	0.02	0.67	0.96
zpTau181	1.05	0.42	0.94	1.17	1.32	< 0.01	1.16	1.51
zBiomarker Factor Score	1.17	0.01	1.03	1.32	1.66	< 0.01	1.43	1.93

	**Transformed LW Scores**							
	Cognitively Decline				Died			
	OR	*P*	lower CI	upper CI	OR	*P*	lower CI	upper CI
zGFAP	1.18	0.01	1.05	1.32	1.31	< 0.01	1.12	1.52
zNfL	1.13	0.14	0.96	1.32	1.71	< 0.01	1.43	2.05
zAβ42/Aβ40	0.99	0.77	0.91	1.07	0.78	0.01	0.65	0.94
zpTau181	1.04	0.50	0.93	1.17	1.32	< 0.01	1.15	1.52
zBiomarker Factor Score	1.18	0.01	1.04	1.35	1.70	< 0.01	1.45	2.00

Note: Each biomarker was included in the model separately.

MODELS INCLUDING CONTROL FOR PLATE

Note for Tables S10 – S12: Runs were done for models with individual neurodegenerative biomarkers as multiple markers would require multiple plate controls. For Aβ42/Aβ40, while most were done on the same plate, 4 were done on different plates and the Aβ40 plate was used as control.
Differences in significant results between these results with plate controls and those in the main body of the paper are highlighted in yellow.

Table S10. Coefficients for the Results on Cognitive Function in Figure 4 and Table S3 (yellow indicates change in significance) when Plates is Controlled

	LASIDAD				HRS			
	b	*P*	lower CI	upper CI	b	*P*	lower CI	upper CI
zGFAP	-0.10	< 0.01	-0.14	-0.06	-0.20	< 0.01	-0.23	-0.16
zNfL	-0.10	< 0.01	-0.13	-0.06	-0.13	< 0.01	-0.16	-0.10
zAβ42/Aβ40	0.04	0.06	-0.00	0.08	-0.03	0.06	-0.06	0.00
zpTau181	0.01	0.77	-0.04	0.05	-0.09	< 0.01	-0.12	-0.05
	zGFAP	zNfL	zAβ42/Aβ40	zpTau181	zGFAP	zNfL	zAβ42/Aβ40	zpTau181
Adj R^2	0.20	0.20	0.19	0.19	0.47	0.45	0.44	0.45

Table S11. ORs for the Results on Dementia in Figure 5 and Table S4 (yellow indicates change) when Plate is Controlled

	LASIDAD				HRS			
	OR	*P*	lower CI	upper CI	OR	*P*	lower CI	upper CI
zGFAP	1.64	< 0.01	1.32	2.05	1.45	< 0.01	1.25	1.68
zNfL	1.38	< 0.01	1.18	1.63	1.14	0.03	1.01	1.29
zAβ42/Aβ40	0.75	0.15	0.51	1.10	1.07	0.91	0.93	1.22
zpTau181	1.43	0.02	1.05	1.95	1.12	0.11	0.98	1.28
	zGFAP	zNfL	zAβ42/Aβ40	zpTau181	zGFAP	zNfL	zAβ42/Aβ40	zpTau181
Pseudo R^2	0.24	0.24	0.21	0.23	0.24	0.23	0.23	0.23
-2Log L	696.27	696.27	696.27	696.27	1087.91	1087.91	1087.91	1087.91

Table S12. ORs for the Results in Figure 6 and Table S5 (yellow indicates change) when Plate is Controlled

	**LASIDAD**							
	Cognitive Decline				Died			
	OR	*P*	lower CI	upper CI	OR	*P*	lower CI	upper CI
zGFAP	1.12	0.35	0.89	1.40	1.53	< 0.01	1.19	1.98
zNfL	1.11	0.63	0.72	1.73	3.67	< 0.01	2.31	5.82
zAβ42/Aβ40	1.01	0.95	0.79	1.28	0.77	0.10	0.56	1.05
zpTau181	0.96	0.58	0.81	1.12	1.00	0.99	0.82	1.21
	zGFAP		zNfL		zAβ42/Aβ40		zpTau181	
Pseudo R^2	0.25		0.31		0.24		0.23	
-2Log L	2281.37		2281.37		2281.37		2281.37	

	**HRS**							
	Cognitive Decline				Died			
	OR	*P*	lower CI	upper CI	OR	*P*	lower CI	upper CI
zGFAP	1.15	0.02	1.02	1.30	1.30	< 0.01	1.11	1.52
zNfL	1.11	0.21	0.94	1.30	1.74	< 0.01	1.44	2.10
zAβ42/Aβ40	0.99	0.74	0.91	1.07	0.81	0.02	0.67	0.97
zpTau181	1.04	0.50	0.93	1.17	1.32	< 0.01	1.15	1.51
	zGFAP		zNfL		zAβ42/Aβ40		zpTau181	
Pseudo R^2	0.18		0.19		0.18		0.18	
-2Log L	5760.47		5760.47		5760.47		5760.47	
